# Effect of Electron-Withdrawing Substituents on Raman Spectra of Diaryl-BTBT Derivatives

**DOI:** 10.3390/ijms27115088

**Published:** 2026-06-04

**Authors:** Olga D. Parashchuk, Liya A. Poletavkina, Mikhail V. Vener, Ivan V. Dyadishchev, Yuriy N. Luponosov, Oleg V. Borshchev, Sofia N. Korchkova, Sergey A. Ponomarenko, Dmitry Y. Paraschuk, Andrey Y. Sosorev

**Affiliations:** 1 Faculty of Physics, Lomonosov Moscow State University, Leninskie Gory 1/2, Moscow 119991, Russia; olga_par@physics.msu.ru (O.D.P.); paras@physics.msu.ru (D.Y.P.); 2 Enikolopov Institute of Synthetic Polymeric Materials, Russian Academy of Science, Profsoyuznaya 70, Moscow 117393, Russia; l.poletavkina@ispm.ru (L.A.P.); dyadischev_iv@ispm.ru (I.V.D.); luponosov@ispm.ru (Y.N.L.); borshchev@ispm.ru (O.V.B.); ponomarenko@ispm.ru (S.A.P.); 3 Kurnakov Institute of General and Inorganic Chemistry of the Russian Academy of Sciences, Leninskii Prosp. 31, Moscow 119991, Russia; mikhail.vener@gmail.com; 4 Faculty of Fundamental Physical and Chemical Engineering, Leninskie Gory 1/2, Moscow 119991, Russia; s.korchkova@ispm.ru

**Keywords:** organic semiconductors, DFT, dynamic disorder, low-frequency vibrations, fluorination, BTBT, Raman spectroscopy, intermolecular interactions, molecular crystals

## Abstract

Low-frequency (LF, ν ≤ 200 cm^−1^) vibrational modes of crystalline organic semiconductors are of particular interest because they significantly affect charge transport in these materials. Herein, we study LF vibrations of [1]benzothieno[3,2-b][1]benzothiophene (BTBT) substituted by phenyls, (per)fluorophenyls or pyridyls using the synergy of Raman spectroscopy and (periodic) DFT calculations. The LF spectra for the compounds with electron-withdrawing (fluorine or nitrogen) atoms differ significantly in the band positions and intensities from those for diphenyl-substituted BTBT, whereas the high-frequency (HF, ν > 200 cm^−1^) spectra are quite similar for all the compounds studied, excluding the perfluorophenyl-substituted BTBT. We found that Ph-BTBT-Ph counterparts containing one electron-withdrawing atom per aryl ring show significantly lower LF Raman intensity compared to the parent compound. The LF intensity decrease is attributed to the suppression of intermolecular motions by the stronger electrostatic interactions. The unexpected LF intensity increase for the perfluorophenyl-substituted BTBT can be ascribed to strong dynamic disorder induced by easier torsion of phenyls with respect to the BTBT core, which also results in the deterioration of the π-conjugation revealed in the HF Raman spectra. We anticipate that the established structure–property relationships will contribute to the rational design of crystalline organic semiconductors towards controlled dynamic disorder and high charge mobility.

## 1. Introduction

Organic electronics is a rapidly growing area of science and technology [1,2,3]. However, except for organic light-emitting diodes (OLEDs), organic electronic devices have not been commercialized yet. To be competitive with the inorganic ones, these devices need better efficiency and operational stability. For example, for organic field-effect transistors (OFETs), organic semiconductors (OSCs) with high charge-carrier mobility exceeding 1 cm^2^V^−1^s^−1^ (a typical value for amorphous silicon, the workhorse of thin-film inorganic electronic devices) are required [3].

Electron–phonon interaction (EPI) strongly impacts charge-carrier mobility, *μ*, in OSCs [1,4,5,6,7]. If a charge carrier is delocalized over several molecules and undergoes (partially) coherent motion, non-local (off-diagonal) EPI converts the dynamic disorder in atomic coordinates [8]—their fluctuation as a result of thermal motion—into dynamic disorder in the intermolecular transfer integrals, *J* [9] and deteriorates charge transport [4,5,6,7]. Thermally activated low-frequency (LF, *ν* ≤ 200 cm^−1^) vibrations, which are mostly intermolecular, are the main culprits for the dynamic disorder, and they are assumed to limit *μ* in many high-mobility OSCs [4,5,6]. Thus, to maximize *μ*, revealing the relationships between the molecular/crystal structure and dynamic disorder is required to search for or design OSCs with low dynamic disorder. Several strategies including the addition of alkyl [10] and electron donating/withdrawing [11,12] substituents were suggested for this purpose. Although the effect of alkyl substituents on LF vibrations and dynamic disorder was addressed in a few reports [9,10,13,14], the corresponding impact of electron donating/withdrawing substituents has been much less studied. Specifically, dynamic disorder can be suppressed by them via changing the crystal packing motif from a layered to a brickwork one [11]. However, whether the dynamic disorder can be suppressed directly, i.e., retaining the molecular packing, is an open issue.

To establish the possible relationship between the molecular structure and dynamic disorder, a series of compounds with slightly varied molecular structure would be beneficial—for instance, those with the same conjugated core and various substituents that have a weak effect on the molecular packing motif. Good candidates for such a series are the [1]benzothieno[3,2-b][1]benzothiophene (BTBT) derivatives, which are among the most promising OSCs for p-channel OFETs since for some of them the reported hole mobility exceeds 5 cm^2^V^−1^s^−1^ [15,16,17]. The versatility of chemical structures realized by the addition of various aromatic and aliphatic substituents to the BTBT core is another reason for their popularity in organic electronics [2,18,19,20]. For instance, diphenyl-substituted BTBT and its derivatives are promising in organic light-emitting transistors (OLETs) [21] and organic light-emitting transistors (OLETs) [17,22,23] because of the high oscillator strength of S_1_-S_0_ transition [15,21]. The substitution of Ph-BTBT-Ph with electron-accepting fluorine atoms can facilitate electron injection and change the conductivity type from the hole one to electron or ambipolar one [22].

Dynamic disorder can be assessed experimentally using Raman spectroscopy [24,25,26,27]. The ability of Raman spectroscopy to probe dynamic disorder stems from the fact that the Raman intensity for a given vibrational mode is determined by modulation of the material polarizability, *α*, by this mode [28,29,30,31,32,33,34]. In the LF range, which is associated mainly with large-amplitude relative motions of molecules contributing to non-local EPI [35,36,37,38,39], total Raman intensity correlates with the relative dispersion of transfer integrals, *σ_J_*^2^/*J*^2^ [25,27]—a measure of dynamic disorder. On the contrary, in the HF range, the Raman intensity is mainly related to the local EPI associated with the reorganization energy λ [24,40,41,42,43,44]. We have recently introduced the spectroscopically available quantity *R* as the ratio of Raman intensities, *I*, integrated over LF and HF ranges:

(1) R=1kT∫LFIdν/∫HFI/νdν,

where *k* is the Boltzmann constant and *T* is the absolute temperature. This quantity is the so-called ‘descriptor of dynamic disorder’ that allows us to estimate the relative standard deviation of the fluctuating transfer integrals *σ_J_*/*J*:

(2) $$\frac{\sigma_{J}}{J}\sim \frac{\sqrt{R\lambda }\;}{E_{g}-{h\nu }_{p}},$$

where *E*_g_ is the optical gap, *ν*_p_ is the pump frequency, and *h* is the Planck constant. Given that *E_g_* and *λ* are similar and *ν_p_* is the same for the compounds from the given series, a larger *R* (i.e., larger relative LF intensity) indicates stronger dynamic disorder. Although this relation is semiquantitative, *R* was shown to correlate well with *σ_J_*/*J* for various OSCs [11,26,27,45] and was further expanded to probing the dynamic disorder and compaction in nucleic acids [46,47]. Using the *R* ratio, we have shown that stronger electrostatic interactions [25] induced by the addition of electron-withdrawing or -donating substituents [11] can dramatically suppress the dynamic disorder. It is particularly beneficial to combine LF Raman spectroscopy with periodic DFT calculations [26,48,49,50] to shed light on the types of motion in crystalline OSCs associated with intensive experimental LF Raman bands and to explain the effect of the changes in the molecular structure on the Raman spectra.

**Figure 1:**
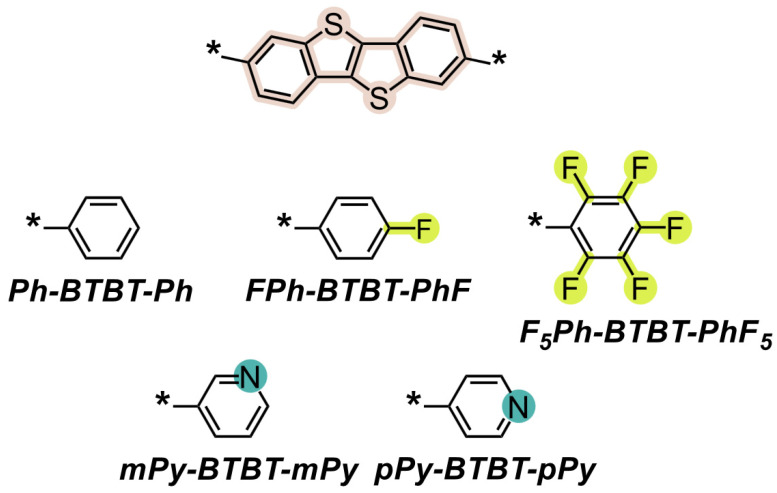
Chemical structures of the compounds studied.

In this study, we apply Raman spectroscopy to address LF vibrations and speculate on the corresponding dynamic disorder in a series of BTBT derivatives: diphenyl-substituted BTBT and its counterparts containing one (FPh-BTBT-PhF) or five (F_5_Ph-BTBT-PhF_5_) electron-withdrawing fluorine substituents per phenyl ring, as well as two dipyridyl-substituted BTBT derivatives with electron-withdrawing nitrogen atoms in the aryl ring either in para- (pPy-BTBT-pPy) or meta- (mPy-BTBT-mPy) position to the BTBT core. The chemical structures of the compounds studied are shown in Figure 1. (Periodic) DFT calculations allowed us to assign the experimental Raman bands, to enlighten the corresponding types of motion, and to suggest an explanation for the observed spectral changes. The correspondence between the calculated spectra for single molecules and crystals, as well as between the spectra calculated using different functionals, is analyzed. The HF spectra for the compounds are rather similar, except for F_5_Ph-BTBT-PhF_5_, which shows an increased intensity in the range 200–800 cm^−1^. In contrast to the HF spectra, the LF spectra for the compounds studied differ significantly in band positions and intensities. Importantly, electron-accepting substituents decrease the LF intensity for all the compounds except for F_5_Ph-BTBT-PhF_5_; this can be ascribed to the suppression of dynamic disorder via the increased strength of intermolecular interactions. The unexpected LF intensity increase for F_5_Ph-BTBT-PhF_5_ is rationalized in terms of weakened π-conjugation between the BTBT core and perfluorophenyl rings resulting in higher freedom of the core libration. The revealed structure–property relationships are expected to facilitate the rational molecular design of OSCs with controlled dynamic disorder.

## 2. Results and Discussion

### 2.1. HF Spectra

**Figure 2:**
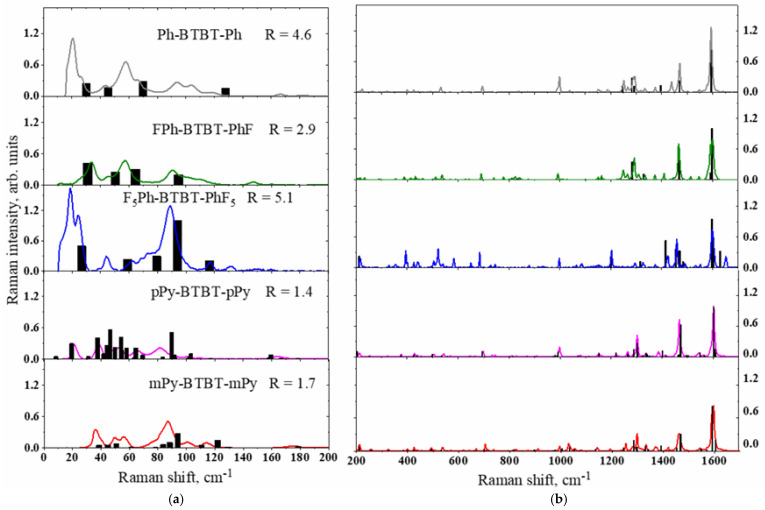
Experimental values (lines) and values calculated using periodic DFT (bars) Raman spectra for the compounds studied: (**a**) LF range, (**b**) HF range. The *R* values are extracted from the experimental spectra by using Equation (1). The *y* axis is the same in panels (**a**) and (**b**), allowing for a direct comparison of LF and HF intensities.

**Figure 3:**
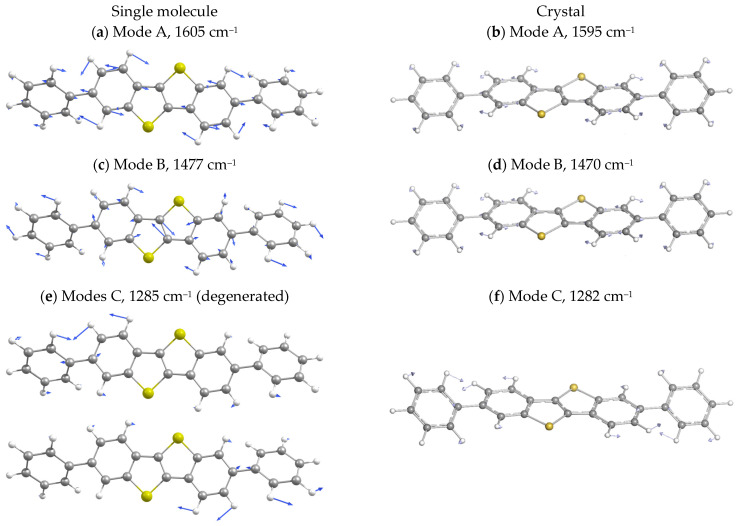
Atomic displacements (arrows) for the strongest Raman-active modes in the HF region of the Ph-BTBT-Ph molecule (**left**) and crystal (**right**). Carbon atoms are shown in grey, sulfur ones in yellow, and hydrogen ones in white.

**Figure 4:**
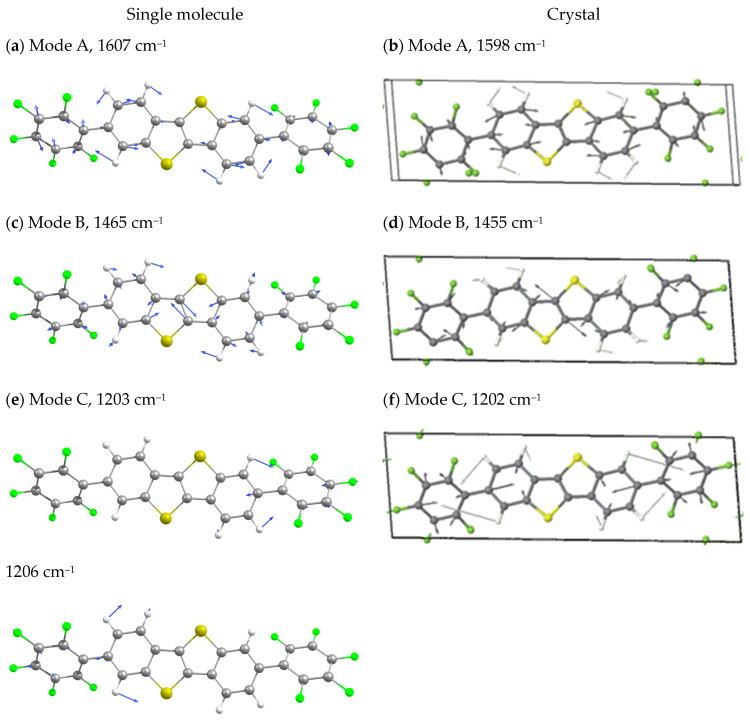
Atomic displacements (arrows) for the strongest Raman-active modes in the HF region of the F_5_Ph-BTBT-PhF_5_ molecule (**left**) and crystal (**right**) obtained at PBE-D3/6-31g(d,p) level. Carbon atoms are shown in grey, sulfur ones in yellow, hydrogen ones in white, and fluorine ones in green.

Figure 2 presents Raman spectra for Ph-BTBT-Ph and its counterparts with fluorine or nitrogen atoms. As shown in Figure 2b, the HF spectra are very similar in the typical range of intensive modes of π-conjugated oligomers, 1000–1700 cm^−1^. The strongest vibrational bands are observed at 1590, 1475 and 1292 cm^−1^. The results of DFT calculations for single (isolated) molecules and periodic DFT calculations for crystals are collated in Table 1, showing the frequencies and relative intensities of these bands. The additional data from single-molecule calculations (frontier orbital patterns, bond orders, optical gaps and oscillator strengths, polarizabilities, reorganization energies, etc.) are provided in the Supporting Information (SI), Section S1. Figure 3 and Figure 4 show the calculated atomic displacements for the three strongest Raman modes (corresponding to the most intense experimental bands) of crystalline Ph-BTBT-Ph and F_5_Ph-BTBT-PhF_5_, respectively; those for the other compounds studied are shown in Figures S4–S7, S11 and S12. The frequencies (see Table 1 and Table S2) and atomic displacements (see Figure 3 and Figure 4) for the HF modes calculated for isolated molecules and crystals are generally similar since these modes are intramolecular. However, the DFT results for crystals (i.e., obtained using periodic DFT) correspond to the experiment better than those for single molecules, most probably because the equilibrium molecule geometry in crystals differs from that of single molecules. Below, we use the experimental wavenumbers for the bands unless otherwise stated.

As follows from our calculations, the experimental band of Ph-BTBT-Ph at 1590 cm^−1^ is associated with the spreading/contraction of benzene rings of the BTBT core towards a quinoid form (mode A, see Figure 3a,b). Such an assumption is in line with Ref. [51], where a similar vibrational pattern was observed for phenyl rings of the conjugated thiophene-phenylene oligomers. This band shifts to a higher wavenumber (~1600 cm^−1^) for derivatives with para-substituents, namely FPh-BTBT-Ph, F_5_Ph-BTBT-PhF_5_ and pPy-BTBT-pPy. One and five fluorine atoms per phenyl ring result in similar shifts in the band to 1599 cm^−1^. For pPy-BTBT-pPy, this shift reaches its maximum so that the band peaks at 1602 cm^−1^. On the other hand, for mPy-BTBT-mPy, the frequency of this band is nearly unaltered, in line with weak conjugation with the meta-positioned atoms [52]. In mPy-BTBT-mPy and FPh-BTBT-PhF, the band at 1590 cm^−1^ splits into two bands; for F_5_Ph-BTBT-PhF_5_, an additional band at ~1650 cm^−1^ is observed. The relative intensity of the 1590 cm^−1^ Ph-BTBT-Ph Raman band decreases for all counterparts with electronegative heteroatoms by 20–30%.

The frequency increase and intensity decrease in mode A with the introduction of electron-withdrawing atoms can be explained as follows. Both single-molecule DFT calculations (SI, Table S3) and X-ray data (Table S4) show that the torsion angle between the aryl ring and the BTBT core increases with fluorination of the phenyl ring and its substitution for pyridyls, reaching its maximum for F_5_Ph-BTBT-PhF_5_. In turn, the stronger torsion of (per)fluorinated phenyls and pyridyls can be explained by changes in the electrostatic potential (ESP) (Figure S9): the hydrogens bound to carbon atoms at 2 and 6 positions (adjacent to the BTBT core) of aryl rings have larger positive Mulliken charges (Figure S10) for substituted molecules and repel the hydrogen atom of the BTBT core more strongly. For F_5_Ph-BTBT-PhF_5_, another factor facilitates torsion: the larger size of fluorine atoms as compared to hydrogen ones induces steric hindrance between the fluorine atoms and the hydrogen atoms of the BTBT core [22]. The increased repulsion between the BTBT hydrogen atoms and the (fluoro)phenyl or pyridyl rings hinders the vibration of the BTBT core and increases in the effective force constant, facilitating the frequency increase. The increased torsion decreases the conjugated length, lowering the intensity of mode A [24,53,54]. In addition, for F_5_Ph-BTBT-PhF_5_, the bond orders in aryl substituents are much lower than in the other compounds studied (Figure S2), probably indicating their less aromatic character (corroborated by the chemical shifts for the protons placed in the centers of the rings, see Table S1) in line with Refs. [55,56,57]. The lesser aromaticity of perfluorophenyls could also contribute to their exclusion from the π-conjugated system: the HOMO density on them is considerably lower than on aryls in the other compounds studied (Figure S1). The bond orders for BTBT cores are nearly the same for the compounds studied (Figure S2), indicating that the aromaticity of the core remains unchanged within the series.

The band at 1470 cm^−1^ corresponds to a collective vibration of the molecule with the largest displacements observed for the central bond of the thienothiophene fragment and hydrogens of the phenyl rings (mode B, see Figure 3c,d). This assignment is in line with Ref. [51], where the bond length alternation for thiophene rings was observed at a similar frequency. This band shifts to the lower wavenumbers with any of the substituents. The maximum shift is for F_5_Ph-BTBT-PhF_5_, which can be explained by the substitution of hydrogen atoms (which are involved into this vibration in Ph-BTBT-Ph) for more massive fluorine atoms. The meta- and para- positions of nitrogen atoms in the pyridine rings again affect the shift in this band in different ways (see Table 1). The para-position of fluorine atoms in the phenyl rings and nitrogen atoms in the pyridine rings result in similar and small shifts in the Raman band and similar increases in the corresponding intensities, while the compound with meta-pyridine shows a strong shift in the B band but small changes in its intensity.

The band at 1292 cm^−1^ corresponds to the spreading/contraction of single bonds linking the phenyl or pyridyl rings to the BTBT moiety and the tilting of C–H bonds of these rings, as shown in Figure 3e,f (mode C). This band shifts to the higher frequencies for pyridine-BTBT (by 10 cm^−1^ for pPy-BTBT-pPy and 5 cm^−1^ for mPy-BTBT-mPy) and nearly does not shift for fluorinated FPh-BTBT-PhF. But for F_5_Ph-BTBT-PhF_5_, this band is attenuated, and another band has higher intensity, namely that at 1200 cm^−1^ (see Figure 4e,f).

Importantly, for F_5_Ph-BTBT-PhF_5_, the intensities of several Raman bands in the range of 200–800 cm^−1^ are significantly increased in comparison with the other derivatives (see Figure 2b). This can be explained by the increased torsional angle between the phenyl rings and the BTBT core (see SI, Tables S2 and S3), which decreases the effective conjugation length. Modes A-C, which modulate the conjugation length and hence are stronger in compounds where it is longer [54], lose their intensity in F_5_Ph-BTBT-PhF_5_ as compared to its counterparts, making the other bands relatively more intensive. This is in line with the decrease in intensity for mode A with respect to modes B and C for F_5_Ph-BTBT-PhF_5_ (see Table S2). Note that for the BTBT core, the modes in the 200–800 cm^−1^ range have intensities comparable to those in the 1000–1700 cm^−1^ range (SI, Figure S18), and hence perfluorination of the phenyl rings partially excludes them from the conjugated system, nearly reducing the latter to the BTBT core.

### 2.2. LF Spectra

Experimental LF spectra of the compounds, in contrast to the HF ones, are significantly different, both in band positions and in band intensities (see Figure 2). The Ph-BTBT-Ph spectrum consists of at least six modes and can be divided into three parts with boundaries at 35 cm^−1^ and 80 cm^−1^. Four intense bands peak at 21, 58, 94 and 105 cm^−1^; the former two bands are the strongest ones and have shoulders. The spectrum resembles that for a popular OSC Ph-BTBT-10 [58], whose molecular structure is close to that of the compounds studied: this BTBT derivative has a phenyl substituent at one terminal and a decyl substituent at the other one.

The LF spectrum for FPh-BTBT-PhF is very similar to that of Ph-BTBT-Ph: intense bands with wavenumbers above 40 cm^−1^ are nearly the same for the two compounds. However, the lowest-frequency (and strongest) band for Ph-BTBT-Ph shifts to the higher frequency in FPh-BTBT-PhF (30 cm^−1^) and considerably decreases in intensity. The F_5_Ph-BTBT-PhF_5_ spectrum significantly differs from the two abovementioned spectra. It has two intense bands at 18 and 88 cm^−1^, while the band at ~60 cm^−1^ is weaker and probably shifted, becoming a shoulder of the 88 cm^−1^ band. For pyridyl-substituted pPy-BTBT-pPy and mPy-BTBT-mPy, there are five and four bands of comparable intensities, respectively. These bands are shifted for mPy-BTBT-mPy as compared to pPy-BTBT-pPy to higher frequencies so that the lowest- and the highest-frequency LF bands have ~20 cm^−1^ larger wavenumbers for the former compound. The band at 87 cm^−1^ is the most pronounced in mPy-BTBT-mPy; for pPy-BTBT-pPy, the intensities of the different LF bands are similar.

Importantly, the (relative) LF intensity for all the studied compounds with nitrogen or fluorine atoms (except F_5_Ph-BTBT-PhF_5_) decreases dramatically in experiments with respect to that of Ph-BTBT-Ph, so that *R* decreases from 4.5 for Ph-BTBT-Ph to 1.4 for pPy-BTBT-pPy (see Figure 2). For F_5_Ph-BTBT-PhF_5_, the LF intensity increases with *R* = 5.1. The compounds have similar *E_g_* values and reorganization energies (Table S2) and, as a result, the Raman-based estimation of the dynamic disorder obtained using Equation (2), (*σ_J_*/*J*)*_Raman_*, shows a similar trend to *R* (Table S7 and Figure S19). Hence, according to Ref. [25], the dynamic disorder could decrease for the compounds with one fluorine or one nitrogen atom per aryl ring but increase for the compound with perfluorinated phenyls. Calculation and detailed analysis of the dynamic disorder is the subject of our separate upcoming paper.

To support our experimental data, we referred to our DFT calculations for crystals. Table 2 shows the wavenumbers of the highest-intensity calculated LF modes for Ph-BTBT-Ph and their tentative assignment. These modes are associated with the motion of the molecules as a whole, namely librations around the corresponding axes, hybridized with intramolecular motion [59]―torsion and bending of phenyl rings connected with the BTBT core. The intramolecular LF mode at ~60 cm^−1^ is also predicted in single-molecule calculations; see SI, Figure S3a. In all LF modes, the atoms of the thienothiophene fragment are practically not displaced, whereas those of the terminal phenyl rings usually undergo the strongest displacements.

**Figure 5:**
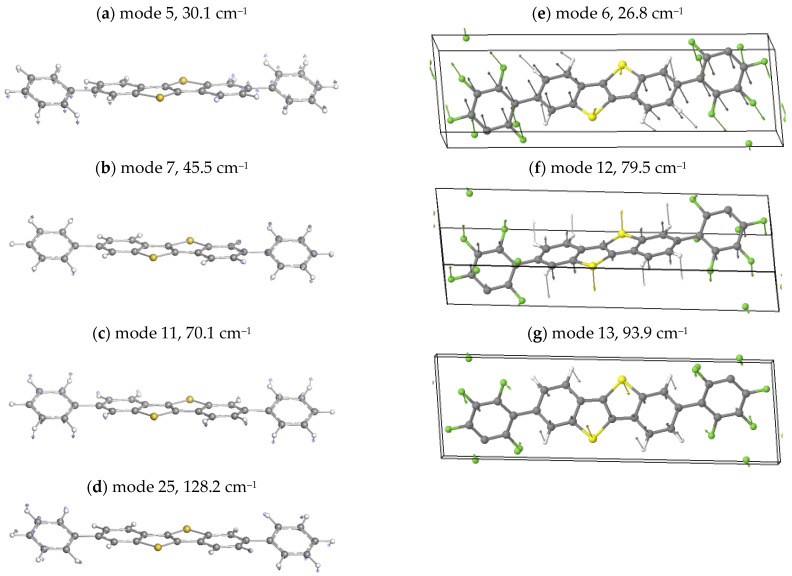
Atomic displacements (arrows) for the selected Raman-active LF modes for the two crystals studied. Ph-BTBT-Ph: (**a**) mode 5, (**b**) mode 7, (**c**) mode 11, (**d**) mode 25. F_5_Ph-BTBT-PhF_5_: (**e**) mode 6, (**f**) mode 12, (**g**) mode 13. Atom colors are the same as for Figure 4.

The lowest-frequency mode at 30 cm^−1^ is a libration around the short molecular axis, *L_y_*, see Figure 5a. The second lowest-frequency mode at 46 cm^−1^ is a complex motion, assigned to libration around the axis normal to the BTBT plane, *L_z_*, combined with the rotation of the terminal phenyls around the long molecular axis, *R_x_*, see Figure 5b. The third LF mode at 70 cm^−1^ is a libration around the long molecular axis, see Figure 5c. The fact that *L_y_* and *L_z_* have lower *ν* values than *L_x_* is reasonable, since the moments of inertia of the molecule around the short axis and around the axis normal to the molecular plane are the largest, and hence the frequency of the corresponding librations should be the lowest [59]. The assignment of the third band (at ~70 cm^−1^) to *L_x_* is in agreement with the polarized Raman data for Ph-BTBT-10 [58]. These assignments are also in line with that for the BTBT crystal, whose molecular structure and packing is close to that of Ph-BTBT-Ph: the modes at 90 and 47 cm^−1^ in BTBT were assigned to *L_x_* and *L_z_*, respectively [25]. Finally, the highest-frequency intense LF mode at 128 cm^−1^ has large intramolecular contribution and can be presented as rotation (tilting) of the phenyl rings around the short molecular axis, *R_y_*, while the BTBT core remains immobile (see Figure 5d).

The lowest-frequency band is the strongest in the experimental LF spectrum of Ph-BTBT-Ph and hence contributes most significantly to the overall LF intensity and *R* descriptor. Accordingly, this mode is expected to contribute noticeably to dynamic disorder. The atomic displacements for the corresponding mode, derived from periodic DFT calculations in crystal, are presented in Figure 5a,b for Ph-BTBT-Ph and F_5_Ph-BTBT-PhF_5_ and in Figures S13–S15 for the other crystals; the corresponding wavenumbers and the relative intensities are summarized in Table S5. In all the crystals studied, this mode can be assigned to libration around the short axis, *L_y_*; the atoms of the BTBT fragments for this mode are displaced much more weakly than those of the terminal phenyls (pyridyls). Importantly, for FPh-BTBT-PhF, an increase in frequency by ~2 cm^−1^ is observed as compared with Ph-BTBT-Ph, in line with the experiment (although the experimental shift is stronger). The calculations also reproduce the difference in the wavenumber of the lowest-frequency mode between pPy-BTBT-pPy and mPy-BTBT-mPy: for the former compound, it is the lowest, and for the latter it is the highest among the crystals studied. Another important mode is that at 94 cm^−1^ for F_5_Ph-BTBT-PhF_5_, which is the strongest according to the calculations and second strongest in the experiment, and hence could contribute significantly to the dynamic disorder. It corresponds to libration around the long molecular axis (*L_x_*), which is at 70 cm^−1^ for Ph-BTBT-Ph; this shift by 24 cm^−1^ explains the lack of the band at ~60 cm^−1^ in the experimental spectra of the F_5_Ph-BTBT-PhF_5_ crystal, in contrast with Ph-BTBT-Ph and FPh-BTBT-PhF.

**Figure 6:**
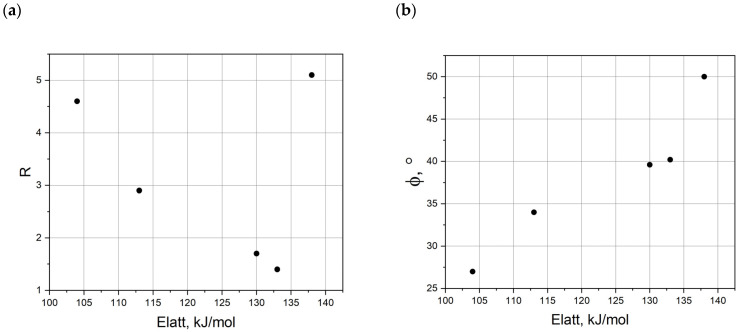
Correlation between the total intermolecular interaction energy *E_latt_* and *R* (**a**) or torsion angle *φ* (**b**).

The decrease in the experimental intensity of the lowest-frequency band for FPh-BTBT-PhF as compared to Ph-BTBT-Ph, as well as the decrease in *R* for all compounds bearing electron-withdrawing atoms (except for F_5_Ph-BTBT-PhF_5_), can be explained by enhancement of the intermolecular interactions. In fact, the total energy of intermolecular interactions, *E_latt_*, calculated from periodic DFT data using the Bader approach (see Equation (S4) in Ref. [26]), increases for Ph-BTBT-Ph counterparts bearing fluorine and nitrogen, as shown in Figure 6a. This can be explained by significantly modified ESP, namely emergence of electronegative areas for the compounds bearing fluorine and nitrogen (see Figure S9). These areas should be involved in electrostatic interactions with electropositive hydrogen atoms of the adjacent molecules, resulting in the increased interaction energy. The higher *E_latt_* for the counterparts with the electron-withdrawing atoms is expected to reduce thermal fluctuations of the atom coordinates, and this explains the decreased *R* for them. For instance, in FPh-BTBT-PhF, the stronger interaction with the molecules from the adjacent molecular layers induced by F∙∙∙H interactions suppresses the amplitude of *L_y_* and hence decreases the lowest-frequency band intensity. Moreover, there is a correlation between *R* and *E_latt_*, with only one outlier: F_5_Ph-BTBT-PhF_5_ (Figure 6a). This correlation is expected since the stronger the interaction, the weaker the dynamic disorder [25].

The unexpected behavior of F_5_Ph-BTBT-PhF_5_ can be explained as follows. The fluorine atoms strengthen intermolecular interactions mostly via perfluorinated phenyls. Meanwhile, the torsion angle *φ* between the BTBT core and the aryl rings increases for those with fluorine and nitrogen atoms and reaches maximum for F_5_Ph-BTBT-PhF_5_ (see Figure 6b). Interestingly, *φ* correlates with *E_latt_*, presumably because stronger intermolecular interactions facilitate torsion of the aryl rings. Accordingly, a larger *φ* decreases the degree of π-conjugation, as revealed from the different HF spectra, namely the weaker relative intensities of A, B and C modes (see above). The weakened π-conjugation between perfluorophenyls and the BTBT core means that the Raman signal for F_5_Ph-BTBT-PhF_5_ comes mainly from the BTBT core, while perfluorophenyls play a role of virtually ‘non-conjugated substituents’, and their interactions with other molecules are not directly linked to the dynamic disorder in the conjugated part. This means that the interaction energy of the latter could be a much lower than *E_latt_* value, explaining the large *R* despite the strong *E_latt_* (Figure 6). Thus, we suppose that only the interactions of the conjugated part of the molecule should be considered when looking for the correlation between the interaction energy and *R*.

Nevertheless, aryl substituents affect the dynamic disorder in the BTBT core indirectly, since the latter is linked to them; the steeper the torsion potential energy curve, the larger the impact of the substituents’ interactions with neighboring molecules on the dynamic disorder of the BTBT core. Phenyl rings’ perfluorination changes the shape of the torsion potential curve (Figure S8); while it was nearly symmetric near equilibrium for Ph-BTBT-Ph, for F_5_Ph-BTBT-PhF_5_ it becomes steeper with a decreasing torsion angle (due to the repulsion between hydrogens and fluorines) and shallower for an increasing torsion angle (probably due to weakened π-conjugation). In the crystal, the torsional angle for F_5_Ph-BTBT-PhF_5_ is larger than for the isolated molecules, so the equilibrium is shifted to the area of softer torsion potential. The net effect of phenyl perfluorination is that the torsional disorder, which can be quantified by the RMSD of the atoms of the BTBT core for *L_x_* (i.e., modes at 79.5 and 93.9 cm^−1^ in calculations), is larger in F_5_Ph-BTBT-PhF_5_ than in Ph-BTBT-Ph (SI, Table S6). Moreover, the electron density at HOMO for F_5_Ph-BTBT-PhF_5_ is shifted out from the long molecular axis to the periphery of the BTBT core (Figure S1a,c), so that (given that LUMO is unchanged, cf. Figure S1f,h) similar torsional disorder in two crystals induces stronger polarizability fluctuation in F_5_Ph-BTBT-PhF_5_. Accordingly, the experimental intensity of the Raman band at 90 cm^−1^ associated with *L_x_* increases significantly, as observed in Figure 2. The stronger torsional disorder for this mode is in line with the structureless photoluminescence spectra of F_5_Ph-BTBT-PhF_5_, while those for Ph-BTBT-Ph and FPh-BTBT-PhF show clear vibronic structure [22].

Noteworthily, for the series of molecules studied, the introduction of electron-withdrawing atoms retains the layered molecular packing motif, in contrast to Ref. [11], where the substitution resulted in a transformation from the layered packing to the brickwork so that the substituted molecules became squeezed by their neighbors and could hardly librate around the short axis and the axis normal to the molecular plane. Molecular squeezing could be the main reason for the disorder suppression in Ref. [11]; indeed, the intensity of the most intensive Raman mode, *L_y_*, decreased dramatically. Our results reported herein show that the enhancement of the intermolecular interaction via electron-withdrawing substituents could suppress dynamic disorder directly, without the crossover from the layered molecular packing to, e.g., brickwork (with respect to long molecular axis) arrangement. The direct effect of electron-withdrawing atoms on LF Raman spectra and *R*, and―within the framework of Refs. [25,26,27]―on dynamic disorder is most pronounced for FPh-BTBT-PhF, which has a very similar molecular packing motif to Ph-BTBT-Ph: both have a layered structure with herringbone packing within the layer, with a just slightly larger shift along the long axis for the molecules in the same layer for FPh-BTBT-PhF [22].

## 3. Methods

### 3.1. Synthesis and Characterization

The synthesis of FPh-BTBT-PhF and F_5_Ph-BTBT-PhF_5_ is described in our previous paper [22]. The mPy-BTBT-mPy and pPy-BTBT-pPy compounds were synthesized by a Suzuki cross-coupling reaction between 2,7-dibromobenzo[b]benzo[4,5]thieno[2,3-d]thiophene and the corresponding boron-organo pyridine derivatives (Figure S16). The reactions were carried out in a microwave reactor. Following the isolation process, the crude product underwent a purification procedure via recrystallisation. The yields of the target compounds were 61% and 63% for mPy-BTBT-mPy and pPy-BTBT-pPy, respectively. Differential scanning calorimetry curves for the selected compounds are shown in Figure S17.

### 3.2. Calculations

DFT calculations for single molecules were performed using either PBE-D3 (PBE [60] with Grimme’s dispersion correction D3 [61]) or B3LYP-D3 (B3LYP [62] with D3 dispersion correction) functional and 6-31G(d,p) basis set. GAMESS (2019.R1.P1.mkl version) package [63,64] was used. For visualization, Chemcraft (https://www.chemcraftprog.com (accessed on 1 December 2022)) [65] and JMol (http://www.jmol.org/ (accessed on 1 December 2022)) [66] programs were used. Crystal structures were obtained from CCDC [67]. The CCDC numbers for them are 837916 (Ph-BTBT-Ph), 2332916 (FPh-BTBT-PhF), 2332917 (F5Ph-BTBT-PhF5), 1907772 (pPy-BTBT-pPy), and 1907770 (mPy-BTBT-mPy). The structures were then optimized, and IR and Raman spectra of the crystals were calculated using the periodic DFT method at the PBE-D3/6-31G** level within Crystal17 Package [68]. PBE-D3/6-31G** provides a grounded trade-off between the accuracy of the relative Raman activities and a reasonable description of the wave numbers of the bands in the considered frequency region; see Tables S1 and S2 in Ref. [26]. Calculations of the Raman intensities were performed using static polarizability approximation. Calculations of the 1H NMR shielding constants were performed using the ORCA 5.0 package [69,70].

### 3.3. Raman

A Raman microscope (inVia, Renishaw, Pliezhausen, Germany) with a 50× objective lens (Leica DM 2500 M, NA = 0.75, Wetzlar, Germany) equipped with a He–Ne laser (RL633, Renishaw) with the power of 17 mW was used. No signs of sample degradation were noticed. The number of runs was chosen depending on the Raman signal intensity. All spectra for the samples were measured at several points and then averaged to increase the signal-to-noise ratio. The LF spectra were recorded in the confocal regime within the range of 10–450 cm^−1^ (series 1) with a built-in double monochromator (NExT, Renishaw). The HF Raman spectra were recorded in the confocal regime within the range of 100–1800 cm^−1^ (series 2) with Rayleigh edge filters. To reconstruct the Raman spectra covering both the LF and HF ranges, the corresponding spectra were stitched [47].

## 4. Conclusions

To conclude, we investigated experimental and calculated Raman spectra for Ph-BTBT-Ph and its four counterparts with electron-withdrawing atoms—fluorine or nitrogen—in the aryl rings. It is shown that the compounds studied have similar HF Raman spectra, except for the molecule with perfluorinated phenyl rings, F_5_Ph-BTBT-PhF_5_, which shows high relative Raman intensities in the range 200–800 cm^−1^—a signature of weakened π-conjugation. In contrast, the LF Raman spectra for the compounds differ strongly. Specifically, for the compounds with electron-withdrawing atoms, the frequency of the lowest-frequency band, assigned to the molecular libration around the short axis, is shifted to higher values. The intensity of the LF range of the Raman spectrum decreases, again except for F_5_Ph-BTBT-PhF_5_. We assign this decrease to the suppression of dynamic disorder due to enhanced electrostatic intermolecular interactions; noteworthily, this occurs without the crossover from the layered to brickwork packing motif. Combining the data from LF and HF Raman spectra, we suggest that for F_5_Ph-BTBT-PhF_5_, the higher intensity of the LF vibrations is associated with the weakened π-conjugation, which facilitates the libration of the BTBT core with respect to perfluorophenyl rings. We anticipate that the relationships revealed between the molecular structure and LF vibrations provide useful tips for the rational design of crystalline organic semiconductors with reduced dynamic disorder and efficient charge transport.

## Figures and Tables

**Table 1:** Experimental vs. calculated (at PBE-D3/6-31g(d,p) level) wavenumbers (in cm^−1^) of the most intensive HF modes in the crystals studied. Calculated values are obtained using DFT for single molecules and crystals. The relative Raman intensity is given in brackets. Similar comparison of calculations for single molecules at B3LYP/6-31G(d,p) level is given in SI, Table S2.

Compound	Mode A			Mode B			Mode C		
	Exp.	Calculated		Exp.	Calculated		Exp.	Calculated	
		Single Molecule	Crystal		Single Molecule	Crystal		Single Molecule	Crystal
Ph-BTBT-Ph	1590 (1.0)	1605 (1.0)	1595 (1.0)	1470 (0.44)	1477 (0.47)	1470 (0.19)	1292 (0.23)	1285 (0.16)	1282 (0.24)
FPh-BTBT-PhF	1599 (1.0) split	1605 (1.0)	1597 (1.0) 1591 (0.14)	1467 (0.85)	1475 (0.51)	1469 (0.40)	1292 (0.55)	1288 (0.24)	1283 (0.35)
F_5_Ph-BTBT-PhF_5_	1599 (1.0)	1607 (1.0)	1598 (0.18)	1459 (0.75) split	1465 (0.38) 1420 (0.42)	1455 (0.07)	1200 (0.48) split	1206 (0.08) 1203 (0.07)	1202 (0.04)
pPy-BTBT-pPy	1602 (1.0)	1596 (1.0)	1602 (1.0)	1468 (0.72)	1471 (0.62)	1474 (0.65)	1302 (0.42)	1288 (0.26)	1301 (0.27)
mPy-BTBT-mPy	1590 (1.0) split	1591 (1.0) 1602 (0.30)	1592 (1.0) 1604 (0.22)	1462 (0.39)	1474 (0.61)	1468 (0.38)	1297 (0.4)	1284 (0.19)	1284 (0.23)

**Table 2:** Wavenumbers (*ν*) and relative Raman activities (*I_rel_*)^a)^ of the selected bands of crystalline Ph-BTBT-Ph with their tentative assignment.

Mode #	*ν*, cm^−1^	*I_rel_* ^a)^	Tentative Assignment
5	30.1	0.22	Libration around the short axis of the molecule (*L_y_*)
7	45.5	0.15	Libration of BTBT core around the axis normal to its plane combined with the rotation of terminal phenyls around the long axis of the molecule (*L_z_* + *R_x_*)
11	70.1	0.25	Libration around the long axis of the molecule (*L_x_*)
25	128.2	0.14	Rotation (tilting) of terminal phenyls around the short axis of the molecule (*R_y_*)

^a)^ The relative intensity of the most intense Raman mode in the theoretical spectrum is 1.0. It is mode # 225, ν = 1595 cm^−1^.

## Data Availability

The original contributions presented in this study are included in the article/Supplementary Material. Further inquiries can be directed to the corresponding author.

## Supplementary Materials

The following supporting information can be downloaded at https://www.mdpi.com/article/10.3390/ijms27115088/s1. References [71,72] are cited in the Supplementary Materials.

## References

[1] Li Y., Coropceanu V., Brédas J.-L., Brédas J.-L., Marder S.R. (2016). The WSPC Reference on Organic Electronics: Organic Semiconductors.

[2] Ostroverkhova O. (2016). Organic Optoelectronic Materials: Mechanisms and Applications. Chem. Rev..

[3] Schweicher G., Garbay G., Jouclas R., Vibert F., Devaux F., Geerts Y.H. (2020). Molecular Semiconductors for Logic Operations: Dead-End or Bright Future?. Adv. Mater..

[4] Troisi A., Orlandi G. (2006). Dynamics of the intermolecular transfer integral in crystalline organic semiconductors. J. Phys. Chem. A.

[5] Fratini S., Mayou D., Ciuchi S. (2016). The Transient Localization Scenario for Charge Transport in Crystalline Organic Materials. Adv. Funct. Mater..

[6] Schweicher G., Olivier Y., Lemaur V., Geerts Y.H. (2014). What Currently Limits Charge Carrier Mobility in Crystals of Molecular Semiconductors?. Isr. J. Chem..

[7] Giannini S., Carof A., Ellis M., Yang H., Ziogos O.G., Ghosh S., Blumberger J. (2019). Quantum localization and delocalization of charge carriers in organic semiconducting crystals. Nat. Commun..

[8] Červinka C. (2025). Computational insights on dynamic disorder in molecular crystals—From electron structure over phonons to thermodynamics. CrystEngComm.

[9] Dettmann M.A., Cavalcante L.S.R., Magdaleno C.A., Moulé A.J. (2023). Catching the Killer: Dynamic Disorder Design Rules for Small-Molecule Organic Semiconductors. Adv. Funct. Mater..

[10] Schweicher G., D’Avino G., Ruggiero M.T., Harkin D.J., Broch K., Venkateshvaran D., Liu G., Richard A., Ruzié C., Armstrong J. (2019). Chasing the “Killer” Phonon Mode for the Rational Design of Low-Disorder, High-Mobility Molecular Semiconductors. Adv. Mater..

[11] Sosorev A.Y., Vener M.V., Kharlanov O., Feldman E.V., Borshchev O.V., Sorokina N.I., Rybalova T.V., Ponomarenko S.A., Paraschuk D.Y. (2023). Efficient Suppression of Dynamic Disorder in Organic Semiconductors via Electron-Donating and -Withdrawing Substituents. J. Phys. Chem. C.

[12] Kumagai S., Ishii H., Watanabe G., Yu C.P., Watanabe S., Takeya J., Okamoto T. (2022). Nitrogen-Containing Perylene Diimides: Molecular Design, Robust Aggregated Structures, and Advances in n-Type Organic Semiconductors. Acc. Chem. Res..

[13] Giannini S., Di Virgilio L., Bardini M., Hausch J., Geuchies J.J., Zheng W., Volpi M., Elsner J., Broch K., Geerts Y.H. (2023). Transiently delocalized states enhance hole mobility in organic molecular semiconductors. Nat. Mater..

[14] Illig S., Eggeman A., Troisi A., Jiang L., Warwick C., Nikolka M., Schweicher G., Yeates S., Yeates Y.H., Anthony J.E. (2016). Reducing dynamic disorder in small-molecule organic semiconductors by suppressing large-amplitude thermal motions. Nat. Commun..

[15] Iino H., Usui T., Hanna J. (2015). Liquid crystals for organic thin-film transistors. Nat. Commun..

[16] Uemura T., Nakayama K., Hirose Y., Soeda J., Uno M., Li W., Yamagishi M., Okada Y., Takeya J. (2012). Band-like transport in solution-crystallized organic transistors. Curr. Appl. Phys..

[17] Fedorenko R.S., Kuevda A.V., Trukhanov V.A., Konstantinov V.G., Sosorev A.Y., Sonina A.A., Kazantsev M.S., Surin N.M., Grigorian S., Borshchev O.V. (2022). Luminescent High-Mobility 2D Organic Semiconductor Single Crystals. Adv. Electron. Mater..

[18] Kadoya T., Imai T., Sano H., Kobayashi Y., Takashima H., Shirakura M., Kojima H., Yamamoto M., Tahara K. (2025). Benzothienobenzothiophene: Recent uses as a transistor material and derivatization for adding new features in device functions. CrystEngComm.

[19] Xie P., Liu T., Sun J., Yang J. (2022). Structures, Properties, and Device Applications for [1]Benzothieno[3,2-b]Benzothiophene Derivatives. Adv. Funct. Mater..

[20] Kataria M., Choi W., Tsutsui Y., Paitandi R.P., Nobuoka M., Omori Y., Sakurai T., Seki S. (2025). Liquid Crystalline [1]Benzothieno[3,2-*b*][1]benzothiophene Semiconductors with Unsymmetrical Thiomethylphenyl Substitution: Synthesis and Charge Transport. Langmuir.

[21] Zhang D., Zhao C., Zheng X., Wu L., Xu J., Zhou L., Wong P.K.J., Zhang W., He Y. (2023). A study on the luminescence properties of high-performance benzothieno[3,2-b][1]benzothiophene based organic semiconductors. Dye Pigment..

[22] Sosorev A.Y., Trukhanov V.A., Skorotetcky M.S., Polyakov R.A., Konstantinov V.G., Dominskiy D.I., Tafeenko V.A., Borshchev O.V., Ponomarenko S.A., Paraschuk D.Y. (2025). Selectively Fluorinated BTBT Derivatives Combining Pronounced Luminescence with Bipolar or Electron Transport. J. Phys. Chem. C.

[23] Fedorenko R.S., Poletavkina L.A., Trukhanov V.A., Kuklin K.N., Balakirev D.O., Dyadishchev I.V., Saratovsky N.S., Bakirov A.V., Ponomarenko S.A., Luponosov Y.N. (2025). Decyloxy-substituted BTBT derivatives for highly efficient and stable thin-film organic (opto)electronic devices. Phys. Chem. Chem. Phys..

[24] Nuraliev M.K., Parashchuk O.P., Tukachev N.V., Repeev Y.A., Maslennikov D.R., Borshchev O.V., Vainer Y., Paraschuk D.Y., Sosorev A.Y. (2020). Toward probing of the local electron–phonon interaction in small-molecule organic semiconductors with Raman spectroscopy. J. Chem. Phys..

[25] Sosorev A.Y., Parashchuk O.P., Tukachev N.V., Maslennikov D.R., Dominskiy D.I., Borshchev O.V., Polinskaya M.S., Skorotetcky M.S., Kharlanov O., Paraschuk D.Y. (2021). Suppression of dynamic disorder by electrostatic interactions in structurally close organic semiconductors. Phys. Chem. Chem. Phys..

[26] Vener M.V., Parashchuk O.D., Kharlanov O.G., Maslennikov D.R., Dominskiy D.I., Chernyshov I.Y., Paraschuk D.Y., Sosorev A.Y. (2021). Non-Local Electron-Phonon Interaction in Naphthalene Diimide Derivatives, Its Experimental Probe and Impact on Charge Carrier Mobility. Adv. Electron. Mater..

[27] Kharlanov O.G., Maslennikov D.R., Feldman E.V., Abashev G.G., Borshchev O.V., Ponomarenko S.A., Vener M.V., Paraschuk D.Y., Sosorev A.Y. (2021). Spectroscopic Assessment of Charge-Carrier Mobility in Crystalline Organic Semiconductors. Adv. Electron. Mater..

[28] Ambrosch-Draxl C., Auer H., Kouba R., Sherman E.Y., Knoll P., Mayer M. (2002). Raman scattering in YBa2Cu3O7:A comprehensive theoretical study in comparison with experiments. Phys. Rev. B.

[29] Knoll P., Ambrosch-Draxl C., Mihailovich D., Ruani G., Kaldis E., Müller K.A. (1995). Proceedings of the International Workshop on Anharmonic Properties of High-Tc Cuprates.

[30] Zhang S.-L. (2012). Raman Spectroscopy and Its Application in Nanostructures.

[31] Scholz R., Gisslén L., Himcinschi C., Vragović I., Calzado E.M., Louis E., Maroto E.S.F., Díaz-García M.A. (2008). Asymmetry between Absorption and Photoluminescence Line Shapes of TPD: Spectroscopic Fingerprint of the Twisted Biphenyl Core. J. Phys. Chem. A.

[32] Lee S.-Y. (1983). Placzek-type polarizability tensors for Raman and resonance Raman scattering. J. Chem. Phys..

[33] Wood S., Hollis J.R., Kim J.-S. (2017). Raman spectroscopy as an advanced structural nanoprobe for conjugated molecular semiconductors. J. Phys. D Appl. Phys..

[34] Jensen L., Zhao L.L., Autschbach J., Schatz G.C. (2005). Theory and method for calculating resonance Raman scattering from resonance polarizability derivatives. J. Chem. Phys..

[35] Salzillo T., Brillante A., Girlando A. (2021). Terahertz Raman scattering as a probe for electron–phonon coupling, disorder and correlation length in molecular materials. J. Mater. Chem. C.

[36] Brillante A., Bilotti I., Della Valle R.G., Venuti E., Girlando A. (2008). Probing polymorphs of organic semiconductors by lattice phonon Raman microscopy. CrystEngComm.

[37] Schweicher G., Das S., Resel R., Geerts Y. (2024). On the importance of crystal structures for organic thin film transistors. Acta Crystallogr. Sect. C Struct. Chem..

[38] Pedron D., Speghini A., Mulloni V., Bozio R. (1995). Coupling of electrons to intermolecular phonons in molecular charge transfer dimers: A resonance Raman study. J. Chem. Phys..

[39] Harrelson T.F., Dantanarayana V., Xie X., Koshnick C., Nai D., Fair R., Nuñez S.A., Thomas A.K., Murrey T.L., Hickner M.A. (2018). Direct probe of the nuclear modes limiting charge mobility in molecular semiconductors. Mater. Horiz..

[40] Vermeulen D., Corbin N., Goetz K.P., Jurchescu O.D., Coropceanu V., McNeil L.E. (2017). Electron-phonon coupling in anthracene-pyromellitic dianhydride. J. Chem. Phys..

[41] Myers A.B. (1996). Resonance Raman Intensities and Charge-Transfer Reorganization Energies. Chem. Rev..

[42] Egolf D.S., Waterland M.R., Kelley A.M. (2000). Resonance Raman Intensity Analysis of the Carbazole/Tetracyanoethylene Charge-Transfer Complex: Mode-Specific Reorganization Energies for a Hole-Transport Molecule. J. Phys. Chem. B.

[43] Masino M., Salzillo T., Brillante A., Della Valle R.G., Venuti E., Girlando A. (2020). Experimental Estimate of the Holstein Electron–Phonon Coupling Constants in Perylene. Adv. Electron. Mater..

[44] Coropceanu V., Cornil J., da Silva Filho D.A., Olivier Y., Silbey R., Brédas J.L. (2007). Charge Transport in Organic Semiconductors. Chem. Rev..

[45] Lu K., Li Y., Wang Q., Wu L., Ren X., Chen X., Liu L., Li Y., Xu X., Zhang Q. (2026). Metallic charge transport in conjugated molecular bilayers. Nat. Electron..

[46] Sosorev A.Y., Parashchuk O.P., Chicherin I.V., Trubitsyn A.A., Trukhanov V.A., Baleva M.V., Piunova U.E., Kharlanov O., Kamenski P., Paraschuk D.Y. (2024). Probing of nucleic acid compaction using low-frequency Raman spectroscopy. Phys. Chem. Chem. Phys..

[47] Sosorev A., Parashchuk O., Kharlanov O., Chicherin I.V., Trubitsyn A.A., Kamenski P.A., Paraschuk D.Y. (2022). Low-Frequency Raman Scattering of Transfer and Ribosomal RNA. JETP Lett..

[48] Chernyshov I.Y., Vener M.V., Feldman E.V., Paraschuk D.Y., Sosorev A.Y. (2017). Inhibiting low-frequency vibrations explains exceptionally high electron mobility in 2,5-difluoro-7,7,8,8-tetracyanoquinodimethane (F_2_-TCNQ) single crystals. J. Phys. Chem. Lett..

[49] Vener M.V., Kharlanov O.G., Sosorev A.Y. (2022). High-Mobility Naphthalene Diimide Derivatives Revealed by Raman-Based In Silico Screening. Int. J. Mol. Sci..

[50] Bedoya-Martínez N., Schrode B., Jones A.O.F., Salzillo T., Ruzié C., Demitri N., Geerts Y.H., Venuti E., Della Valle R.G., Zojer E. (2017). DFT-Assisted Polymorph Identification from Lattice Raman Fingerprinting. J. Phys. Chem. Lett..

[51] Moreno Castro C., Ruiz Delgado M.C., Hernández V., Hotta S., Casado J., Navarrete J.T.L. (2002). Efficiency of the π conjugation in a novel family of α,α′-bisphenyl end-capped oligothiophenes by means of Raman spectroscopy. J. Chem. Phys..

[52] Skorotetcky M.S., Surin N.M., Svidchenko E.A., Pisarev S.A., Fedorov Y.V., Borshchev O.V., Kuleshov B.S., Shaposhnik P.A., Maloshitskaya O.A., Ponomarenko S.A. (2022). Synthesis and Photophysical Properties of Novel Meta-Conjugated Organic Molecules with 1,3,5-Benzene Branching Units. J. Phys. Chem. B.

[53] Agarwal N., Lucotti A., Fazzi D., Tommasini M.M.S., Castiglioni C., Chalifoux W.A., Tykwinski R.R. (2013). Structure and chain polarization of long polyynes investigated with infrared and Raman spectroscopy. J. Raman Spectrosc..

[54] Zerbi G., Castiglioni C., Navarrete J.L., Bogang T., Gussoni M. (1989). A molecular viewpoint of lattice dynamics and spectra of conducting polymers. Synth. Met..

[55] Torres-Vega J.J., Vásquez-Espinal A., Ruiz L., Fernández-Herrera M.A., Alvarez-Thon L., Merino G., Tiznado W. (2015). Revisiting aromaticity and chemical bonding of fluorinated benzene derivatives. ChemistryOpen.

[56] Rosa N., Máximo-Canadas M., Borges I. (2025). Assessing the Aromaticity of Fluorinated Benzene Derivatives Using New Descriptors Based on the Distributed Multipole Analysis (DMA) Partition of the Electron Density. J. Phys. Org. Chem..

[57] Báez-Grez R., Pino-Rios R. (2022). Evaluation of Slight Changes in Aromaticity through Electronic and Density Functional Reactivity Theory-Based Descriptors. ACS Omega.

[58] Ferrari E., Pandolfi L., Schweicher G., Geerts Y.H., Salzillo T., Masino M., Masino M., Venuti E. (2023). Interlayer Sliding Phonon Drives Phase Transition in the Ph-BTBT-10 Organic Semiconductor. Chem. Mater..

[59] Sosorev A.Y., Maslennikov D.R., Kharlanov O., Chernyshov I., Bruevich V.V., Paraschuk D.Y. (2018). Impact of Low-Frequency Vibrations on Charge Transport in High-Mobility Organic Semiconductors. Phys. Stat. Solidi—Rapid Res. Lett..

[60] Perdew J.P., Burke K., Ernzerhof M., Ernzerhof M. (1996). Generalized Gradient Approximation Made Simple. Phys. Rev. Lett..

[61] Grimme S., Ehrlich S., Goerigk L. (2011). Effect of the Damping Function in Dispersion Corrected Density Functional Theory. J. Comput. Chem..

[62] Lee C., Yang W., Parr R.G. (1988). Development of the Colle-Salvetti Correlation-Energy Formula into a Functional of the Electron Density. Phys. Rev. B.

[63] Schmidt M.W., Baldridge K.K., Boatz J.A., Elbert S.T., Gordon M.S., Jensen J.H., Koseki S., Matsunaga N., Nguyen K.A., Su S.J. (1993). General Atomic and Molecular Electronic Structure System. J. Comput. Chem..

[64] Gordon M.S., Schmidt M.W., Dykstra C.E., Frenking G., Kim K.S., Scuseria G.E. (2005). Advances in Electronic Structure Theory: GAMESS a Decade Later. Theory and Applications of Computational Chemistry: The First Forty Years.

[65] CCDC Crystallographic Database. https://www.ccdc.cam.ac.uk/solutions/csd-system/components/csd/.

[66] Chemcraft—Graphical Software for Visualization of Quantum Chemistry Computations. https://www.chemcraftprog.com.

[67] Jmol: An Open-Source Java Viewer for Chemical Structures in 3D. http://www.jmol.org/.

[68] Dovesi R., Erba A., Orlando R., Zicovich-Wilson C.M., Civalleri B., Maschio L., Rerat M., Casassa S., Baima J., Salustro S. (2018). Quantum-mechanical condensed matter simulations with CRYSTAL. WIRE’s Comput. Mol. Sci..

[69] Neese F. (2022). Software update: The ORCA program system, version 5.0. WIRE’s Comput. Mol. Sci..

[70] Stoychev G.L., Auer A.A., Izsák R., Neese F. (2018). Self-Consistent Field Calculation of Nuclear Magnetic Resonance Chemical Shielding Constants Using Gauge-Including Atomic Orbitals and Approximate Two-Electron Integrals. J. Chem. Theory Comput..

[71] Merrick J.P., Moran D., Radom L. (2007). An evaluation of harmonic vibrational frequency scale factors. J. Phys. Chem. A.

[72] Vyas V.S., Gutzler R., Nuss J., Kernab K., Lotsch B.V. (2014). Optical gap in herringbone and π-stacked crystals of [1]benzothieno[3,2-b]benzothiophene and its brominated derivative. CrystEngComm.

