# Supplementary material for: Effect of Electron-Withdrawing Substituents on Raman Spectra of Diaryl-BTBT Derivatives
Source: Int J Mol Sci. 2026 Jun 4;27(11):5088. doi: 10.3390/ijms27115088 (PMC13257697; doi:10.3390/ijms27115088)
Supplement: Supplementary file 1 [file ijms-27-05088-s001.zip › ijms-4269726-supplementary.pdf]

Supporting Information

Effect of electron-withdrawing substituents on Raman spectra of diaryl-BTBT derivatives

O.D. Parashchuk[1], L.A. Poletavkina[2], M. V. Vener[3], I.V. Dyadishchev[2], Yu.N. Luponosov[2], O.V.Borshchev[2], S.N. Korchkova[4], S.A. Ponomarenko[2], D.Yu. Paraschuk[1], A.Yu. Sosorev[1,2]

[1]*Faculty of Physics, Lomonosov Moscow State University, Leninskie Gory 1/2, Moscow 119991, Russia*
[2]*Enikolopov Institute of Synthetic Polymeric Materials, Russian Academy of Science, Profsoyuznaya 70, Moscow 117393, Russia*
[3]*Kurnakov Institute of General and Inorganic Chemistry of the Russian Academy of Sciences, Leninskii prosp. 31, Moscow 119991, Russia*
[4]*Faculty of Fundamental Physical and Chemical Engineering, Lomonosov Moscow State University, Leninskie Gory 1/2, Moscow 119991, Russia*

S1. Calculations details

Single-molecule calculations

HOMO and LUMO patterns

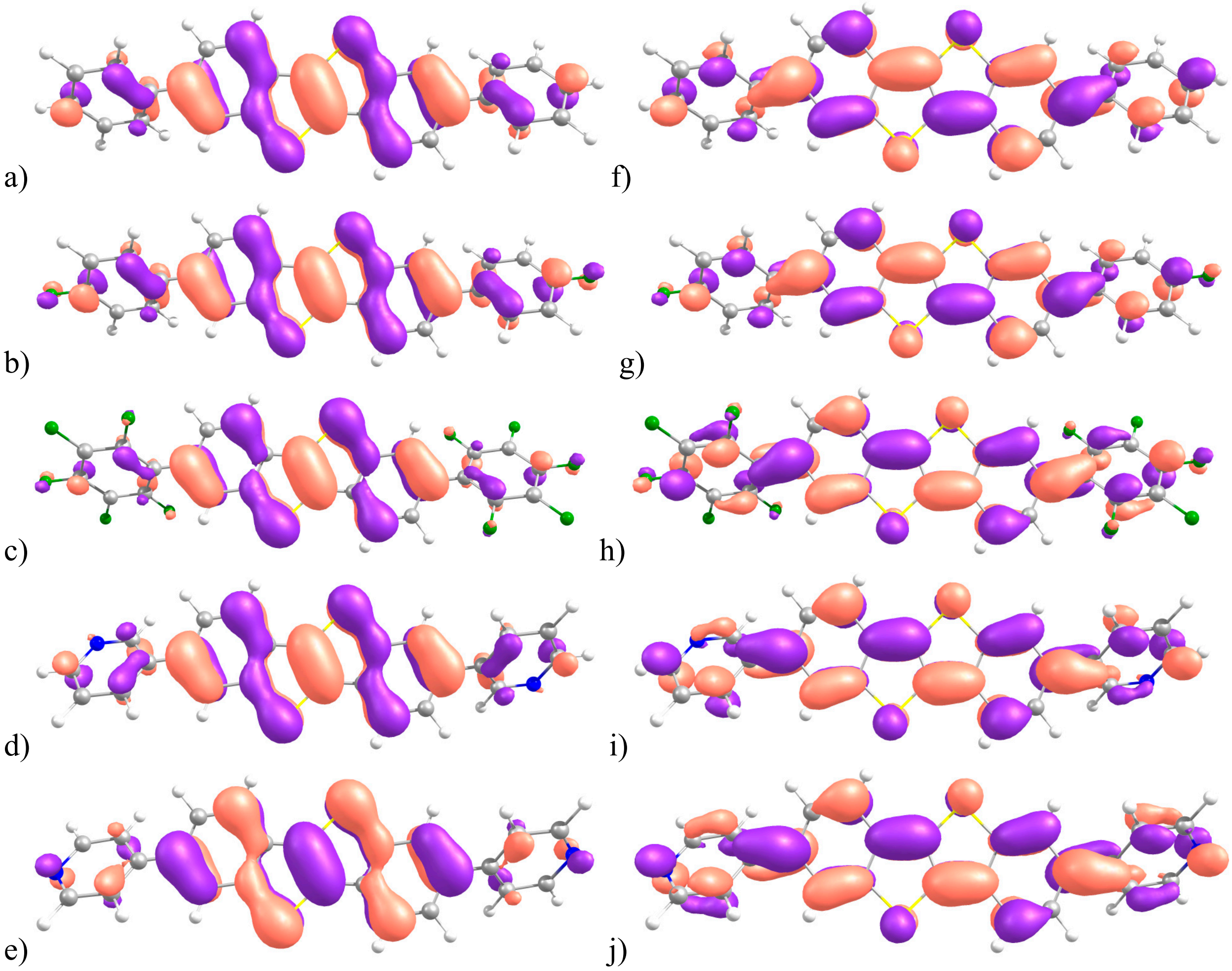

Fig. S1. HOMO (a-e) and LUMO (f-j) for Ph-BTBT-Ph (a,f), FPh-BTBT-FPh (b,g), F_5Ph-BTBT-PhF_5 (c,h), mPy-BTBT-mPy (d,i), pPy-BTBT-pPy (e,j).

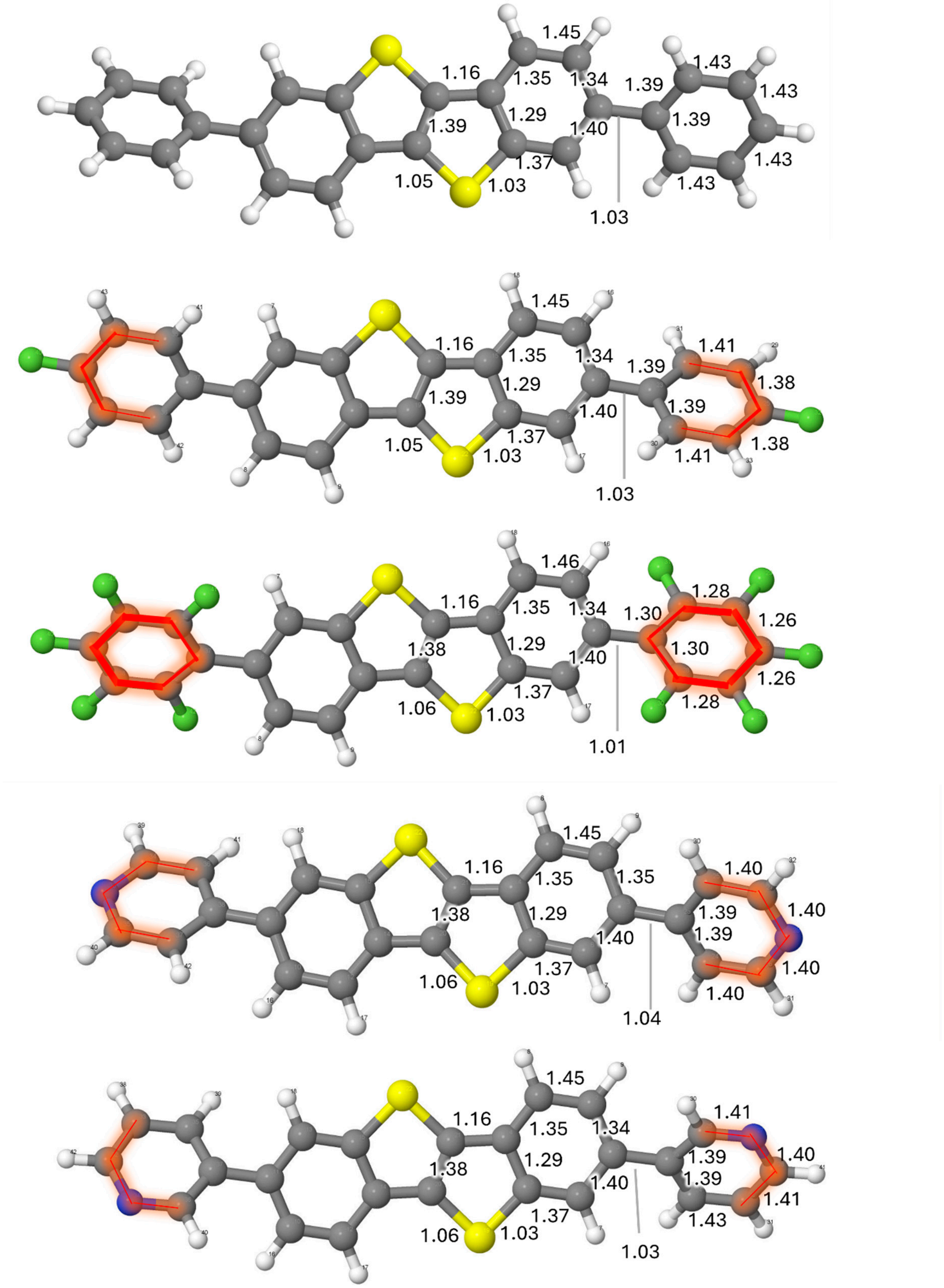

Fig. S2. Bond orders (labels) for the molecules studied obtained at PBE-D3/6-31g(d,p) level. The bonds that show lower bond order than their counterparts in Ph-BTBT-Ph are highlighted with red color.

Table S1. NMR chemical shifts for the protons placed in the centers of the rings for the compounds studied.

	aryl	benzene	thiophene
Ph-BTBT-Ph	-55.1	-16.7	14.9
FPh-BTBT-PhF	-123.5	-17.6	14.9
F_5Ph-BTBT-PhF_5	29.9	-9.9	15.7
pPy-BTBT-pPy	-20.6	-12.5	15.3
mPy-BTBT-mPy	-46.3	-15.3	15.6

Raman-active HF modes

a) 58 cm^{-1}

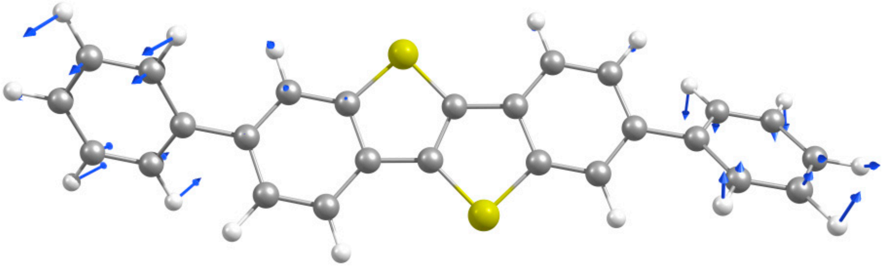

b) 1660 cm^{-1}

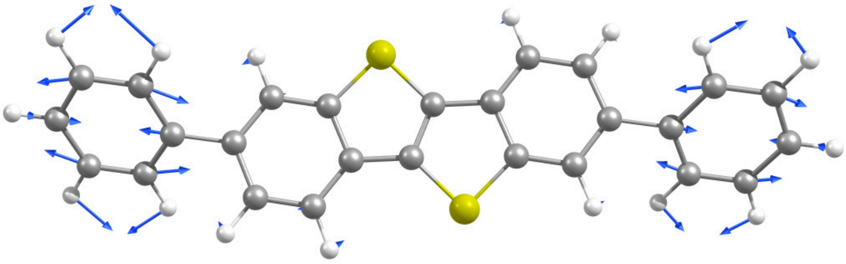

Fig. S3. Atomic displacements for selected HF vibrational modes of Ph-BTBT-Ph single molecules obtained using PBE-D3 functional and 6-31g(d,p) basis set.

a) Mode A (degenerated), 1596 cm⁻¹

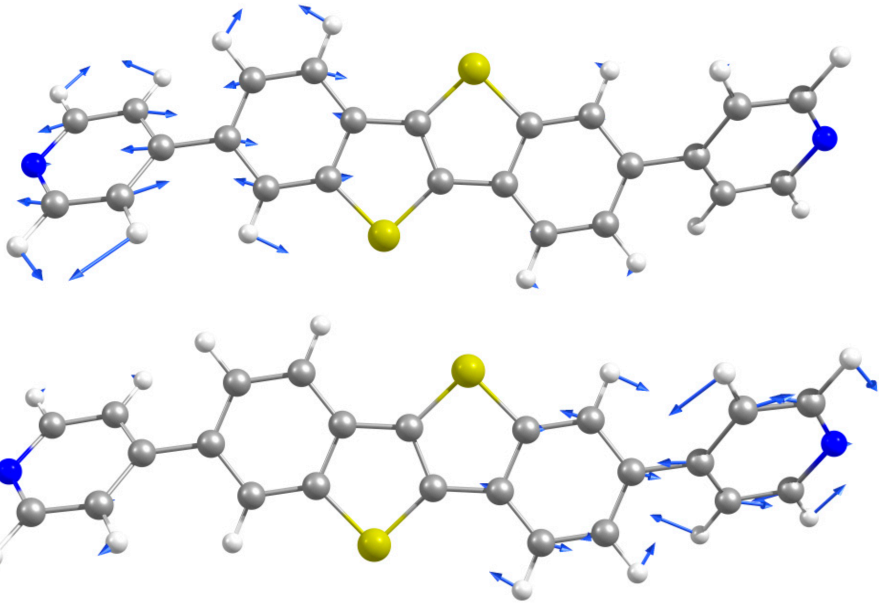

b) Mode B, 1471 cm⁻¹

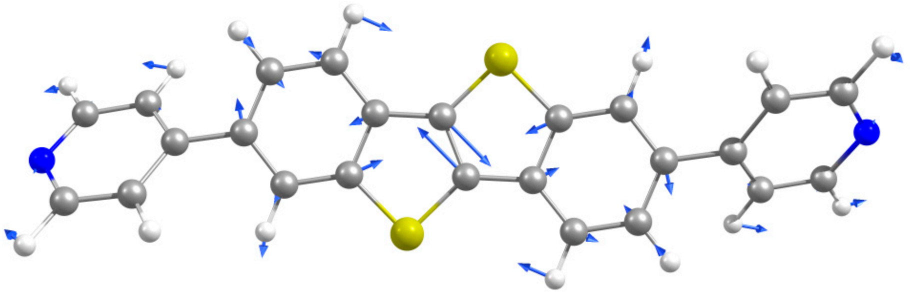

c) Mode C (degenerated) 1288 cm⁻¹

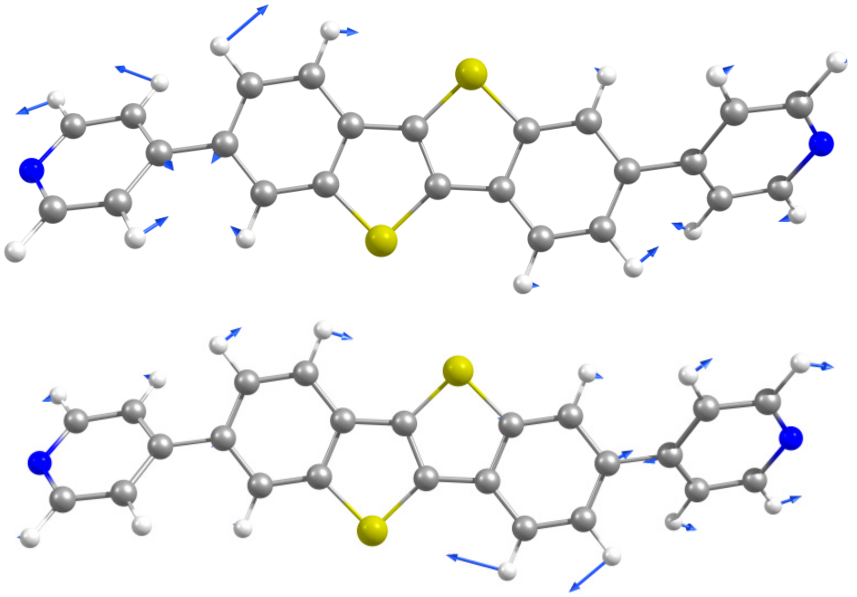

Fig. S4. Atomic displacements for selected HF vibrational modes of pPy-BTBT-pPy single molecules obtained using PBE-D3 functional and 6-31g(d,p) basis set.

a) Mode A (degenerated)

1591 cm^{-1}

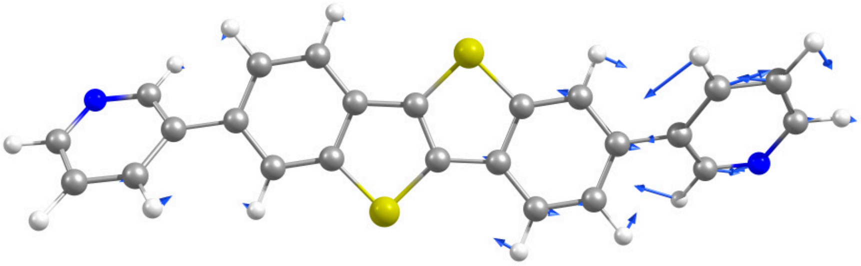

1602 cm^{-1}

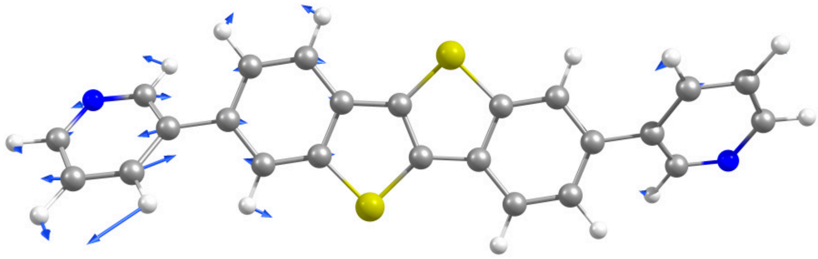

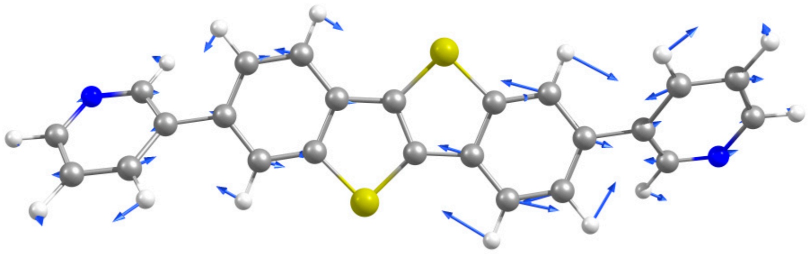

b) Mode B, 1471 cm^{-1}

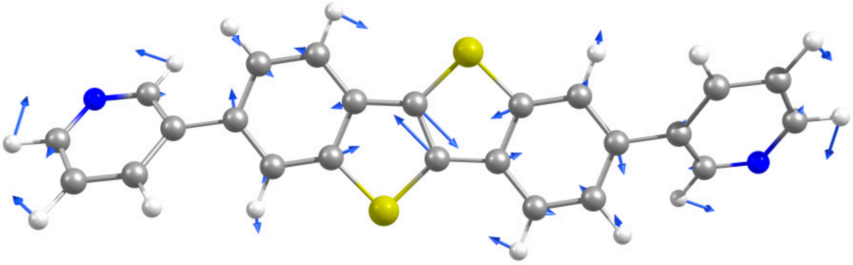

c) Mode C, 1284 cm^{-1}

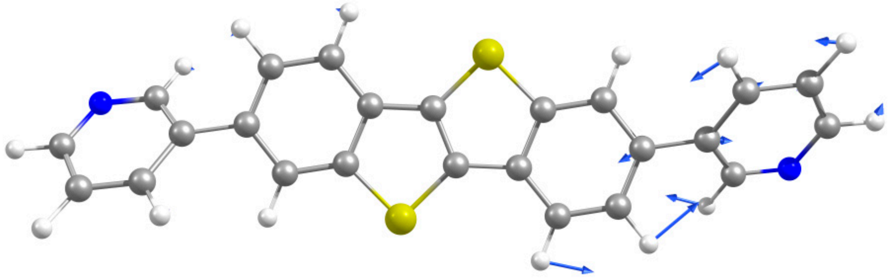

Fig. S5. Atomic displacements for selected HF vibrational modes of mPy-BTBT-mPy obtained using PBE-D3 functional and 6-31g(d,p) basis set.

a) Mode A, 1605 cm^{-1}

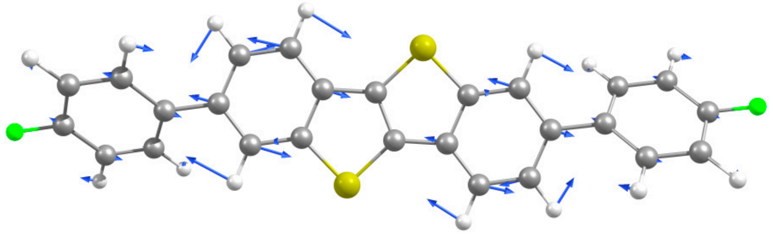

b) Mode B, 1475 cm^{-1}

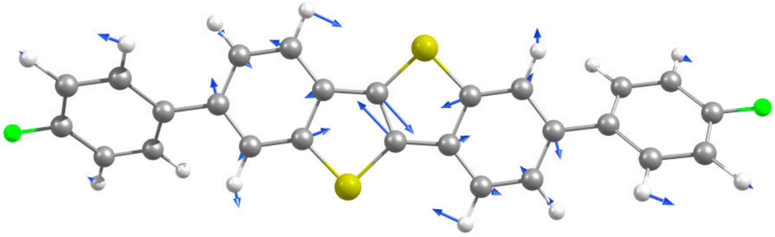

c) Mode 1292 cm^{-1} (degenerated)

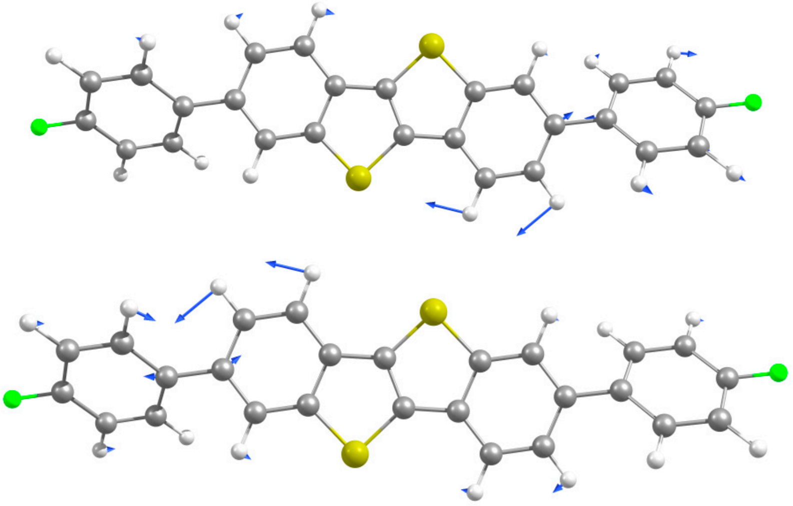

d) 1682 cm^{-1}

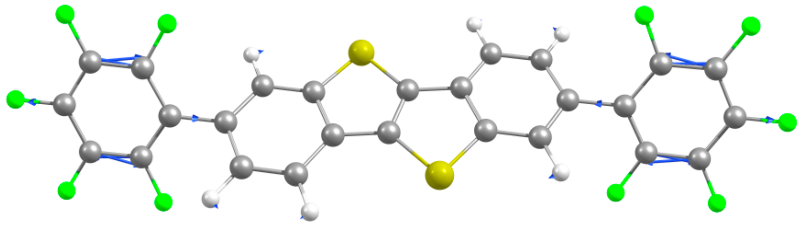

Fig. S6. Atomic displacements for selected HF vibrational modes of FPh-BTBT-PhF single molecules obtained using PBE-D3 functional and 6-31g(d,p) basis set.

a) 21.09 cm^{-1}

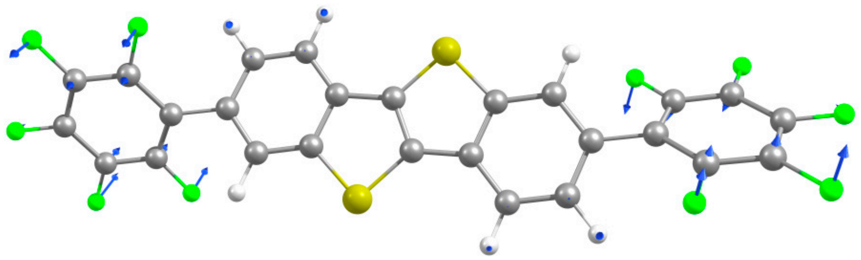

b) 44.54 cm^{-1}

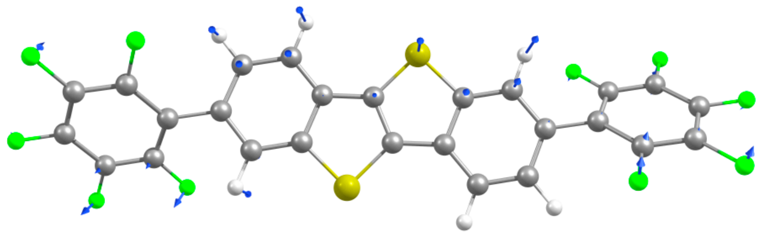

c) 234.48 cm^{-1}

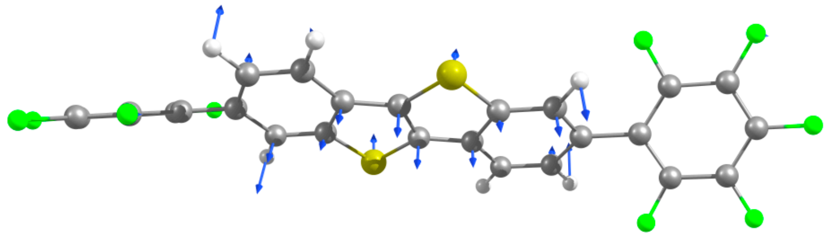

d) 264.72 cm^{-1}

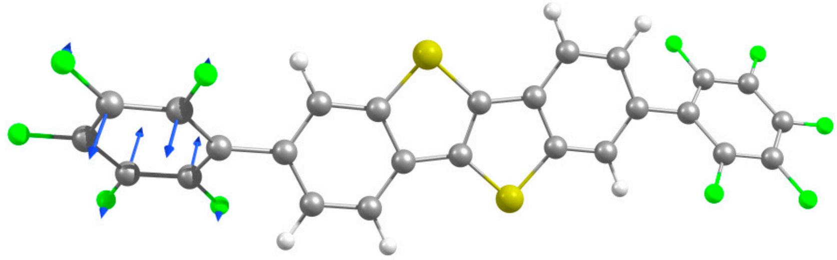

e) 511.89 cm^{-1}

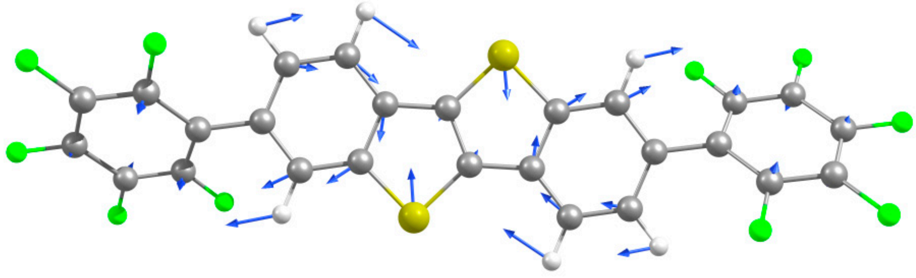

f) 565.69 cm^{-1}

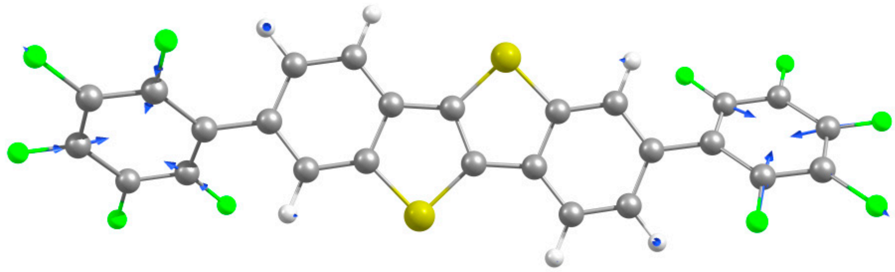

g) 675.08 cm^{-1}

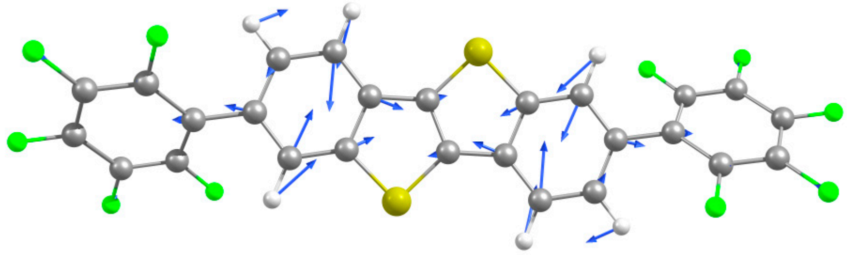

Fig. S7. Atomic displacements for selected HF vibrational modes of F_5Ph-BTBT-PhF_5 obtained using PBE-D3 functional and 6-31g(d,p) basis set.

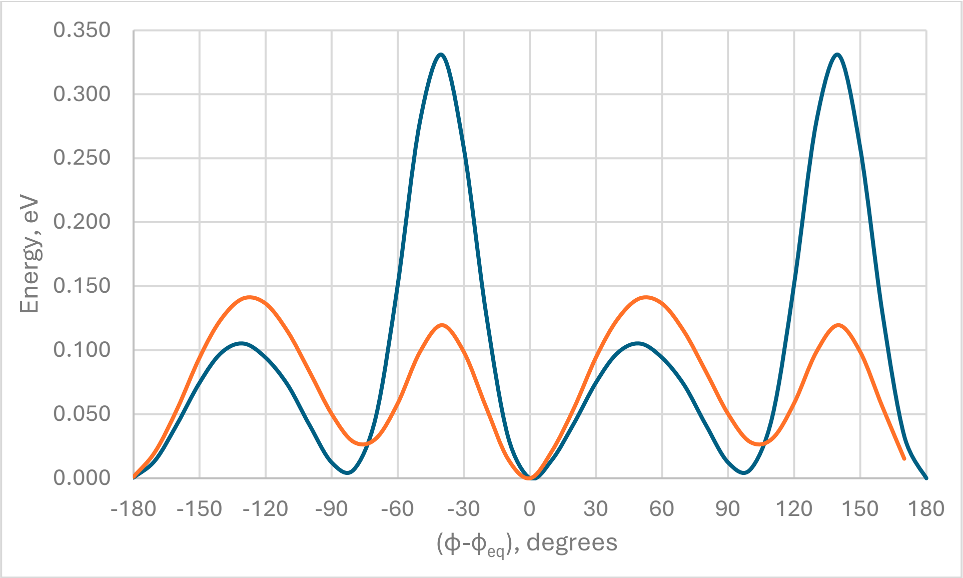

Fig. S8. Potential curves for (perfluoro)phenyl torsion with respect to BTBT core, for Ph-BTBT-Ph (orange) and F_5Ph-BTBT-PhF_5 (blue) single molecules. ϕ_{eq} is the torsional angle at equilibrium geometry.

Electrostatic potential and Mulliken charges

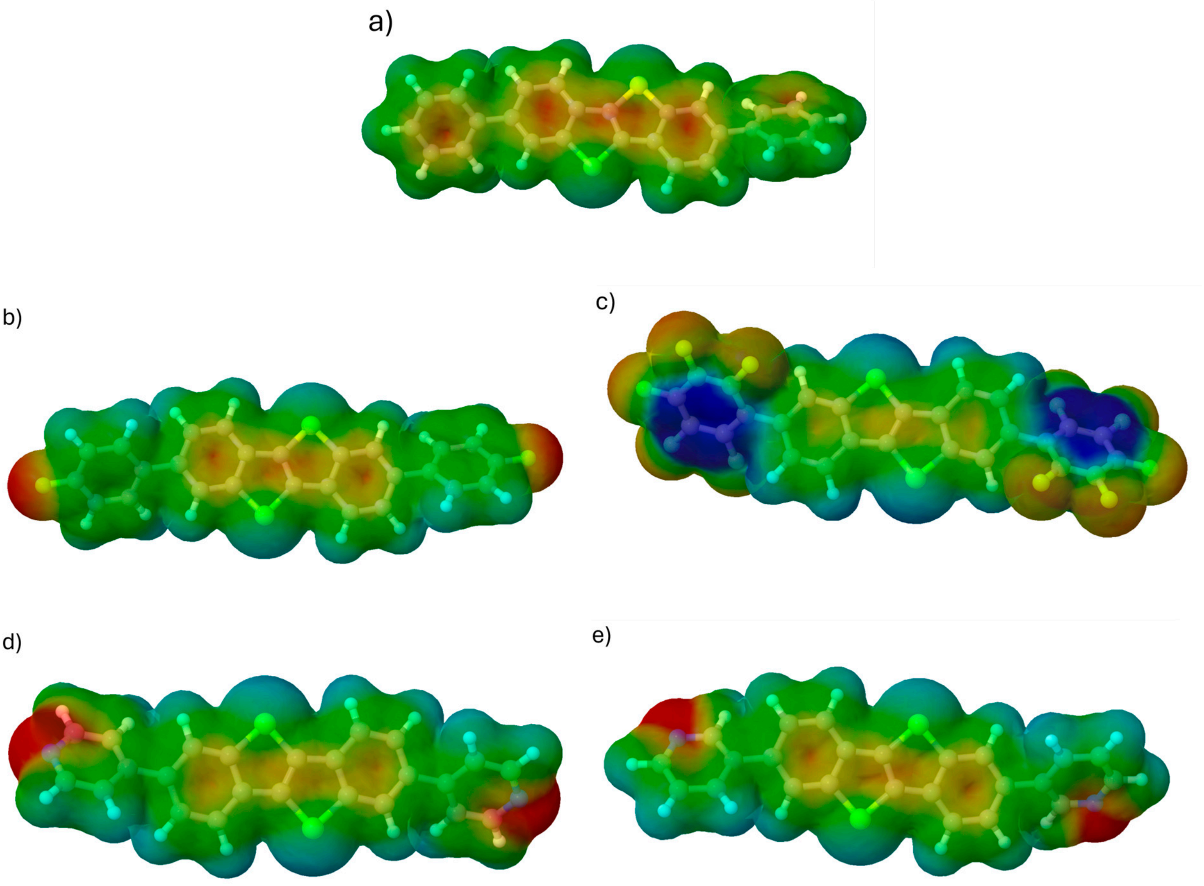

Fig. S9. Electrostatic potentials for the compounds studied: Ph-BTBT-Ph (a), FPh-BTBT-PhF (b), F5Ph-BTBT-PhF5 (c), pPy-BTBT-pPy (d), mPy-BTBT-mPy (e). Red color corresponds to negative potential, blue to the positive one, green depicts slightly electropositive areas and yellow shows slightly electronegative ones.

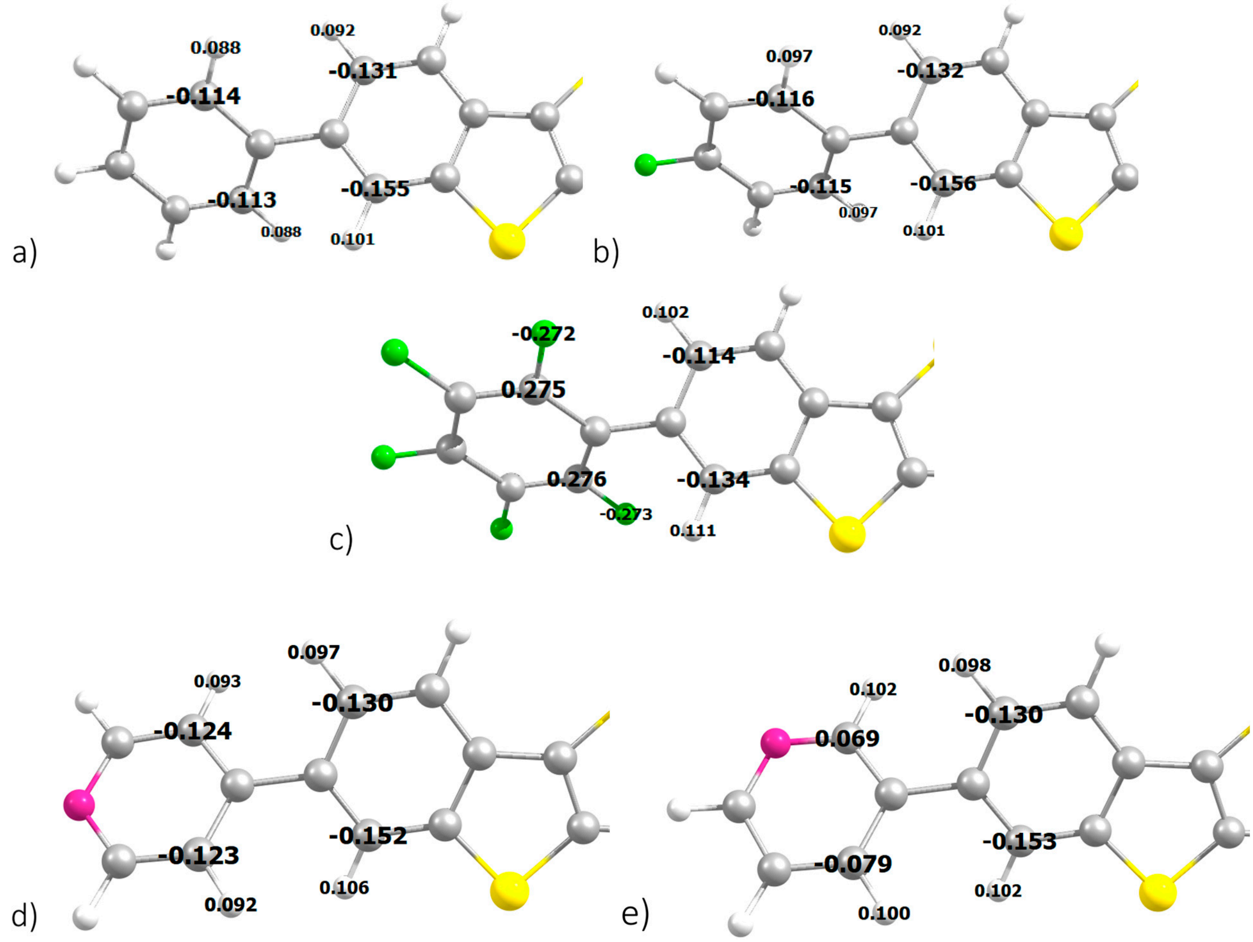

Fig. S10. Mulliken partial charges for the compounds studied: Ph-BTBT-Ph (a), FPh-BTBT-PhF (b), F5Ph-BTBT-PhF5 (c), pPy-BTBT-pPy (d), mPy-BTBT-mPy (e).

Table S2. Experimental vs. calculated wavenumbers (in cm^{-1}) of the most intensive HF modes in the crystals studied. Calculated values are obtained using DFT for the single molecules (at B3LYP-D3/6-31g(d,p) level) and periodic DFT (at PBE-D3/6-31g(d,p) level) for the crystals. The frequencies for single molecules are scaled by 0.97. Such vibrational frequency scaling is a common practice for DFT calculations, and the scaling factor chosen is close to the values of 0.961-0.969 recommended for the B3LYP functional [1]. Relative Raman intensity is given in brackets.

Compound	Mode A			Mode B			Mode C		
	Exp.	Calculations		Exp.	Calculations		Exp.	Calculations	
		single molecules	crystal		single molecules	crystal		single molecules	crystal
Ph-BTBT	1590	1600 (1.0)	1595 (1.0)	1470	1472 (0.33)	1470 (0.19)	1292	1283 (0.18)	1282 (0.24)
FPh-BTBT	1599	1600 (1.0)	1597 (1.0)<br>1591 (0.14)	1467 split	1472 (0.41)<br>1464 (0.20)	1469 (0.40)	1292	1272 (0.18)	1283 (0.35)
F_5Ph-BTBT	1599	1602 (1.0)	1598 (0.18)	1459 split	1456 (0.54)<br>1411 (0.45)	1455 (0.07)	1200	1195 (0.08)	1202 (0.04)
pPy-BTBT	1602	1594 (1.0)	1602 (1.0)	1468	1466 (0.44)	1474 (0.65)	1302	1279 (0.25)	1301 (0.27)
mPy-BTBT	1590 split	1589 (1.0)	1590 (1.0)<br>1597 (0.227)	1462	1467 (0.64)	1468 (0.38)	1297	1284 (0.26)	1284 (0.23)

Table S3. Molecular properties obtained at B3LYP-D3/6-31G(d,p) level for the compounds studied: torsional angle between the BTBT core and aryl rings, φ, HOMO (EH) and LUMO (EL) energies and their difference (ΔE_{HL}), optical gap E_g, oscillator strength for S_0-S_1 transition, exciton binding energy E_{exc}, reorganization energies for hole (λ_h) and electron (λ_e) transfer, and isotropic polarizability (α).

	φ, °	E_H, eV	E_L, eV	ΔE_{HL}, eV	E_g, eV	f	E_{exc}, eV	λ_h, meV	λ_e, meV	α, Å^3
Ph-BTBT-Ph	39.0	-5.33	-1.46	3.87	3.53	1.02	0.34	226	318	54.2
FPh-BTBT-PhF	38.5	-5.41	-1.55	3.86	3.53	1.07	0.33	249	321	54.5
F_5Ph-BTBT-PhF_5	41.9	-5.78	-1.95	3.83	3.48	0.76	0.35	243	330	55.2
pPy-BTBT-pPy	37.3	-5.72	-1.89	3.83	3.34	0.97	0.49	219	327	52.2
mPy-BTBT-mPy	38.9	-5.57	-1.71	3.86	3.39	1.13	0.47	201	381	52.5

Periodic DFT

Table S4. Aryl torsion angles in the crystals of BTBT derivatives with small electron-withdrawing substituents from X-ray data.

Compound	Ph-BTBT-Ph	FPh-BTBT-PhF	F_5Ph-BTBT-PhF_5	pPy-BTBT-pPy	mPy-BTBT-mPy
CCDC entry #	837916	2332916	2332917	1907772	1907770
Torsion angle φ, deg.	27.0	34.3	50.0	40.2	39.6

Comparison of HF modes for single-molecule and periodic DFT calculations

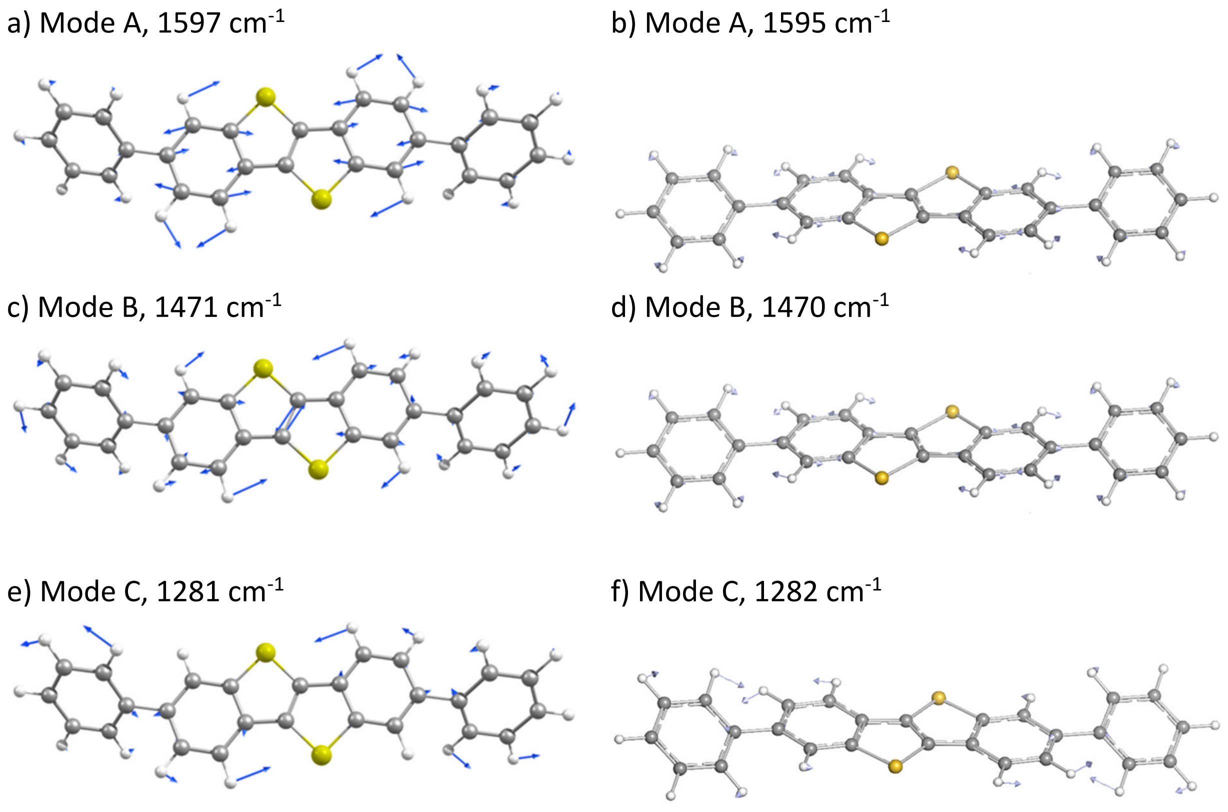

Fig. S11. Atomic displacements for the most Raman-active modes in the HF region of the Ph-BTBT-Ph molecule (left; at B3LYP/6-31g(d,p) level) and Ph-BTBT-Ph crystal (right; at PBE-D3/6-31g(d,p) level).

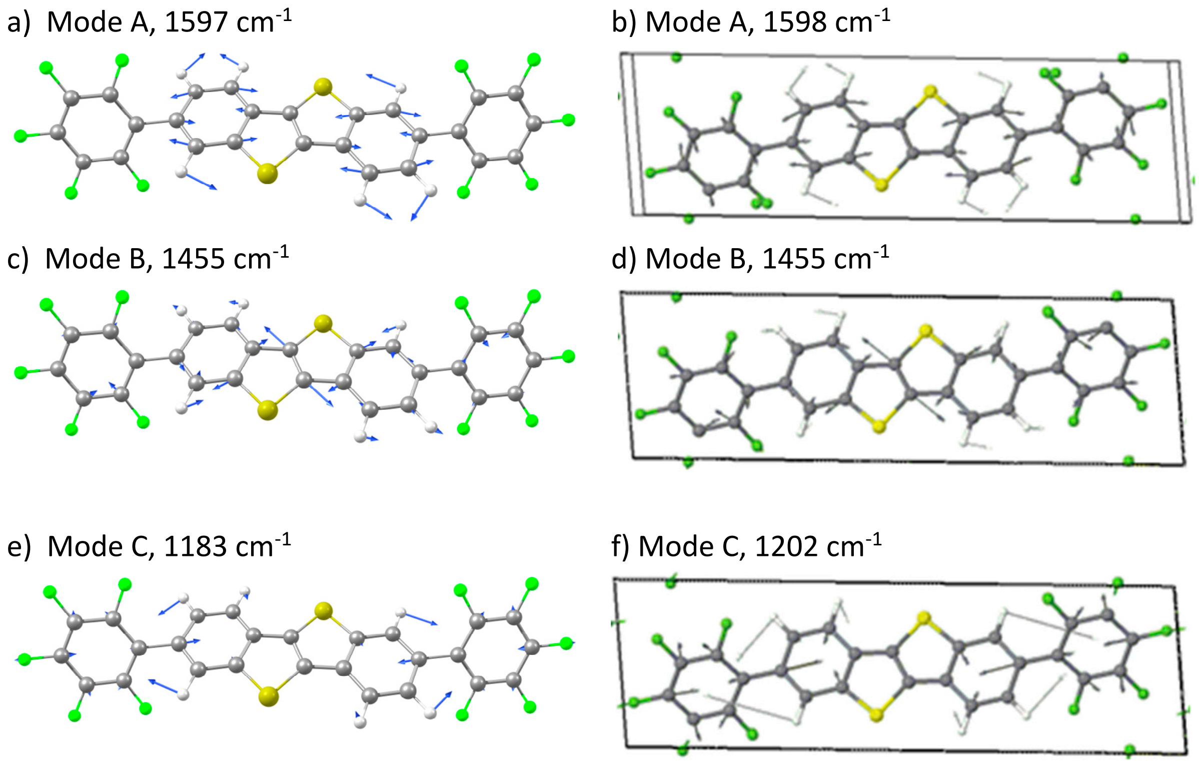

Fig. S12. Atomic displacements for the most Raman-active modes in the HF region of the F_5Ph-BTBT-PhF_5 molecule (left; at B3LYP/6-31g(d,p) level) and F_5Ph-BTBT-PhF_5 crystal (right; at PBE-D3/6-31g(d,p) level).

LF modes

Table S5. Wavenumbers (*v*) and relative Raman activities (I_{rel})[a] of the lowest Raman-active mode in the considered crystals. The atomic displacements of the modes are shown in Figs. 5 and S13-S15.

Crystal	Mode #	ν, cm^{-1}	I_{rel}
Ph-BTBT-Ph	5	30.1	0.22
FPh-BTBT-PhF	4	31.1	0.42
F5Ph-BTBT-PhF5	6	26.8	0.09[b]
pPy-BTBT-pPy	5	20.0	0.31
mPy-BTBT-mPy	5	38.6	0.07

a) The relative activity of the most intense Raman mode in the considered theoretical spectrum is 1.0;

b) Unlike other crystals, the most intense Raman mode of F5Ph-BTBT-PhF5 lies at ~6 cm^{-1}. According to Ref. [2], the region below 20 cm^{-1} is not considered in the calculated Raman spectra.

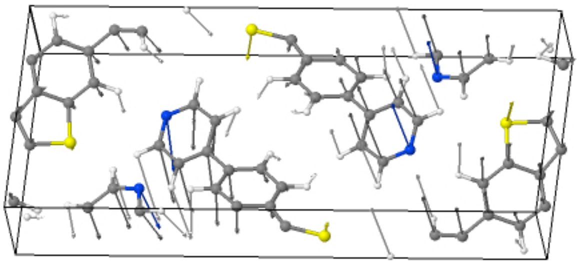

Fig. S13. Atomic displacements of mode 5 (20.0 cm^{-1}) in the pPy-BTBT crystal.

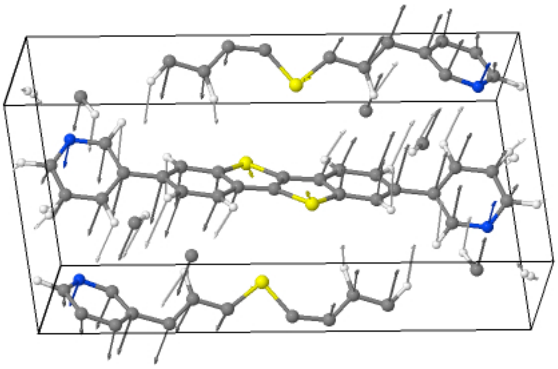

Fig. S14. Atomic displacements of mode 5 (38.6 cm^{-1}) in the mPy-BTBT crystal.

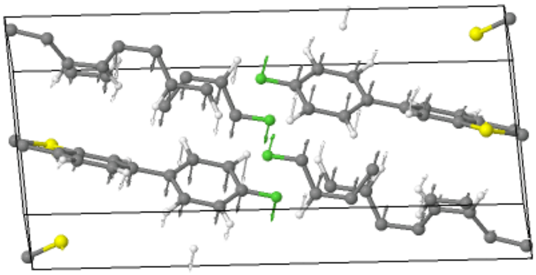

Fig. S15. Atomic displacements of mode 4 (31.1 cm-1) in the F-Ph-BTBT crystal.

Table S6. Root mean square deviation (RMSD, in Å) of atomic coordinates for selected vibrational modes and integrated over all LF modes.

crystal	Ph-BTBT-Ph	FPh-BTBT-PhF	F5Ph-BTBT-PhF5
all atoms, all LF modes	0.29	0.27	0.29 (0.24[a])
Non-hydrogen atoms, all LF modes	0.24	0.22	0.46 (0.23[a])
BTBT core without hydrogens, all LF modes	0.21	0.20	0.22 (0.21[a])
Non-hydrogen atoms, L_x mode	0.06	0.03	0.06
BTBT core without hydrogens, L_x mode[b)]	0.02 (70.1)	0.02 (100)	0.04 (94);<br>0.02 (79.5)

a) The values in brackets are calculated excluding the lowest-frequency mode at ~6 cm^{-1}. According to Ref. [2], the region below 20 cm^{-1} in the calculated vibrational spectrum is not completely reliable.

b) The wavenumber of L_x mode is given in brackets in cm^{-1}.

S2. Synthesis details

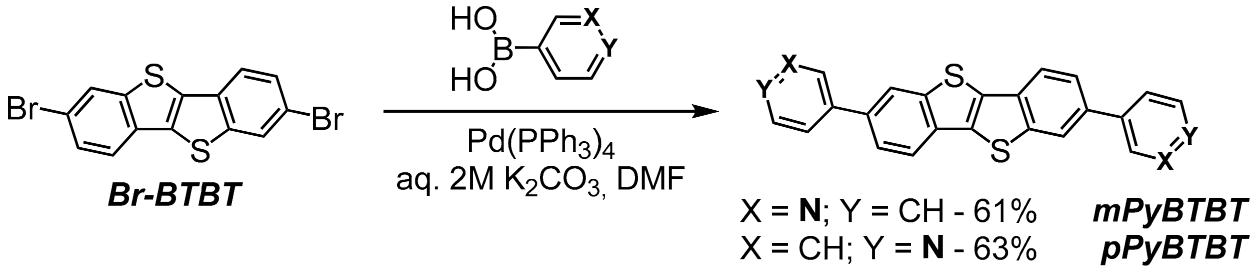

Figure S16. The synthesis scheme of mPy-BTBT- mPy and pPy-BTBT- pPy

The study utilised commercial Ph-BTBT-Ph (Sigma Aldrich). 2,7-dibromobenzo[b]benzo[4,5]thieno[2,3-d]thiophene Br-BTBT-Br was synthesized according to previously described procedure [3]. All reagents were purchased from commercial sources (abcr and Acros) and used without further purification. DMF and toluene were dried and purified according to the known techniques. All reactions were carried out under an inert atmosphere. The chemical structure and purity of the target compounds were confirmed by ^{1}H and ^{13}C NMR spectroscopy, GPC, elemental analysis and MALDI-TOF mass spectrometry. All the synthesized molecules were found to be difficult to solubilize, which limited the taking of ^{13}C NMR spectra.

2,7-di(pyridin-3-yl)benzo[b]benzo[4,5]thieno[2,3-d]thiophene (mPy-BTBT-mPy): To 2,7-dibromo[1]benzothieno[3,2-b][1]benzothiophene (Br-BTBT-Br) (0.60 g, 1.5 mmol), 3-pyridineboronic acid (0.93 g, 7.5 mmol), and tetrakis(triphenylphosphine)palladium(0) $Pd(PPh_3)_4$ (0.10 g, 0.1 mmol) in inert atmosphere were added degassed DMF (48 mL) and 2M K_2CO_3 aqueous solution (11.3 mL). The reaction mixture was stirred at 125 °C in a microwave reactor for 8 h. After completion of the reaction, the cooled reaction mixture was filtered off and washed with water and alcohol. The resulting precipitate was purified by recrystallization in toluene. **mPy-BTBT-mPy** was obtained as a white powder in 61% yield (0.36 g). ^{1}H NMR (300 MHz, $CDCl_3$): δ [ppm] 7.30-7.36 (m, 2H), 7.63 (dd, 2H, J_1 = 1.4 Hz, J_2 = 9.7 Hz), 7.88-7.94 (overlapping peaks, 4H), 8.07 (d, 2H, J = 0.7 Hz), 8.55 (dd, 2H, J_1 = 0.6 Hz, J_2 = 4.6 Hz), 8.88 (d, 2H, J = 1.7 Hz). Anal. calcd (%) for $C_{24}H_{14}N_2S_2$: C, 73.07; H, 3.58; N, 7.10; S, 16.25. Found: C, 73.22; H, 3.81; N, 6.95; S, 16.09. MALDI-TOF MS: found m/z 394.18; calculated for $[M]^+$ 394.06.

2,7-di(pyridin-4-yl)benzo[b]benzo[4,5]thieno[2,3-d]thiophene (pPy-BTBT-pPy): To 2,7-dibromo[1]benzothieno[3,2-b][1]benzothiophene (Br-BTBT-Br) (0.60 g, 1.5 mmol), 4-pyridineboronic acid (0.93 g, 7.5 mmol), and tetrakis(triphenylphosphine)palladium(0) $Pd(PPh_3)_4$ (0.10 g, 0.1 mmol) in inert atmosphere were added degassed DMF (48 mL) and 2M K_2CO_3 aqueous solution (11.3 mL). The reaction mixture was stirred at 130 °C in a microwave reactor for 9 h. After completion of the reaction, the cooled reaction mixture was filtered off and washed with water and alcohol. The resulting precipitate was purified by recrystallization in toluene. **pPy-BTBT-pPy** was obtained as a white powder in 63% yield (0.37 g). ^{1}H NMR (300 MHz, $CDCl_3$): δ [ppm] 7.60 (d, 4H, J = 6.0 Hz), 7.76 (dd, 2H, J_1 = 1.3 Hz, J_2 = 8.3 Hz), 8.01 (d, 2H, J = 8.3 Hz), 8.21 (d, 2H, J = 0.7 Hz), 8.72 (d, 4H, J = 6.0 Hz). Anal. calcd (%) for $C_{24}H_{14}N_2S_2$: C, 73.07; H, 3.58; N, 7.10;

S, 16.25. Found: C, 73.27; H, 3.75; N, 7.13; S, 16.30. MALDI-TOF MS: found m/z 394.27; calculated for $[M]^+$ 394.06.

S3. Thermal properties

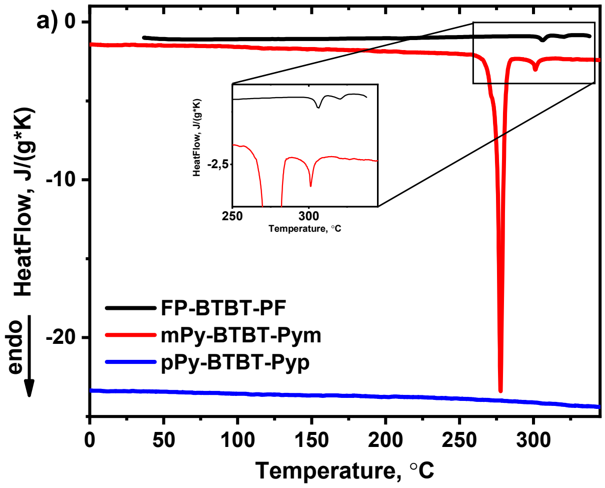

Fig. S17. DSC curves of the first heating for: FP-BTBT-PF, mPy-BTBT-mPy and pPy-BTBT-pPy.

S4. Raman spectra details

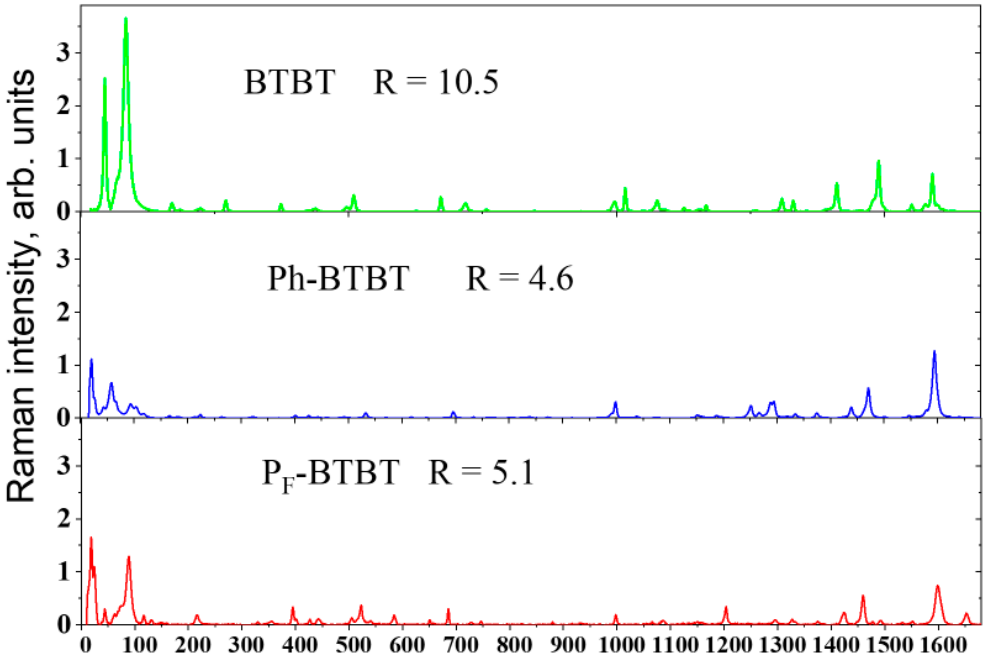

Fig. S18. Experimental Raman spectra for BTBT, Ph-BTBT-Ph and F_5Ph-BTBT-PhF_5.

Table S7. Dynamic disorder in transfer integrals estimated from Raman spectra using Eq. (2), $(\sigma_J/J)_{Raman}$, for the crystals studied. The values of E_g were taken from Table S2, λ was averaged over reorganization energies for holes and electrons from Table S2, and the pump phonon energy in experiment was $h\nu_p$=1.959 eV (633 nm laser).

crystal	$(\sigma_J/J)_{Raman}$, a.u.[a)]
Ph-BTBT-Ph	0.113
FPh-BTBT-PhF	0.091
F_5Ph-BTBT-PhF_5	0.126
pPy-BTBT-pPy	0.071
mPy-BTBT-mPy	0.078

a) Note that $(\sigma_J/J)_{Raman}$ in Eq. (2) are determined up to coefficient, which is similar for various compounds.

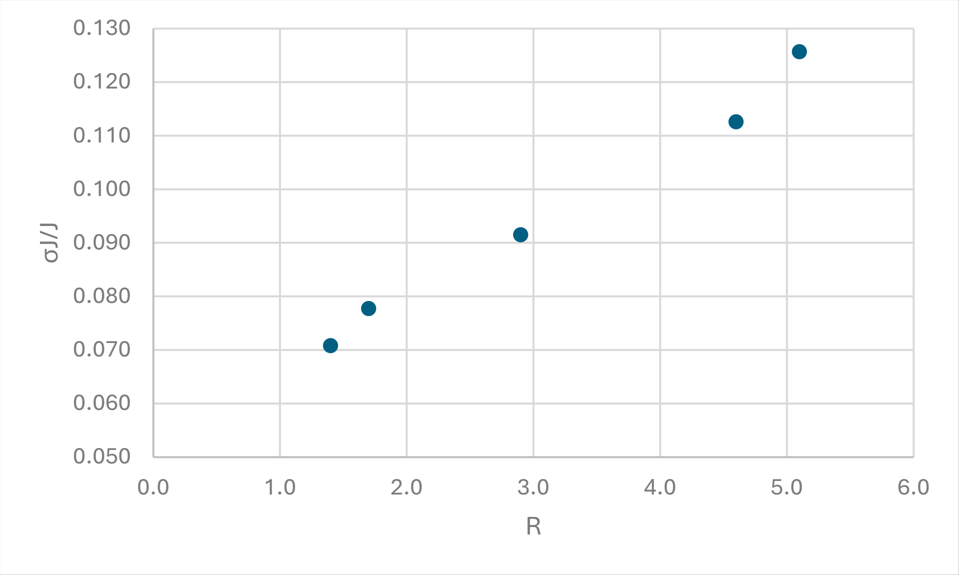

Fig. S19. The correlation between R and $(\sigma_J/J)_{Raman}$.

References

1. J. P. Merrick, D. Moran and L. Radom, J. Phys. Chem. A, 2007, 111, 11683

2. Vener, M. V.; Kharlanov, O. G.; Sosorev, A. Yu. High-Mobility Naphthalene Diimide Derivatives Revealed by Raman-Based In Silico Screening. Int. J. Mol. Sci. 2022, 23, 13305. DOI: 10.3390/ijms232113305

3. Vyas, V. S.; Gutzler, R.; Nuss, J.; Kernab K.; Lotsch, B. V. Optical gap in herringbone and π-stacked crystals of [1]benzothieno[3,2-b]benzothiophene and its brominated derivative, CrystEngComm 2014, 16, 7389–7392, DOI: 10.1039/C4CE00752B.
